# A rotavirus vaccine candidate attenuated by codon deoptimization protects neonatal mice against wild-type virus infection

**DOI:** 10.1371/journal.ppat.1014292

**Published:** 2026-07-07

**Authors:** Zelin Chen, Tomohiro Kotaki, Daisuke Motooka, Shintaro Sato, Yusuke Sakai, Katsuhisa Hirai, Shohei Minami, Takahiro Kawagishi, Yuta Kanai, Takeshi Kobayashi

**Affiliations:** 1 Department of Virology, Research Institute for Microbial Diseases, The University of Osaka, Osaka, Japan; 2 NGS Core Facility, Research Institute for Microbial Diseases, The University of Osaka, Osaka, Japan; 3 Department of Microbiology and Immunology, School of Pharmaceutical Sciences, Wakayama Medical University, Wakayama, Japan; 4 Department of Infectious Disease Pathology, National Institute of Infectious Diseases, Japan Institute for Health Security, Tokyo, Japan; 5 Center for Advanced Modalities and DDS, The University of Osaka, Osaka, Japan; 6 Center for Infectious Disease Education and Research, The University of Osaka, Osaka, Japan; University of Cambridge, UNITED KINGDOM OF GREAT BRITAIN AND NORTHERN IRELAND

## Abstract

Rotavirus infection is a leading cause of acute viral gastroenteritis and diarrhea in infants and young children. Owing to the limited development of effective antiviral therapies, vaccination has become the primary and most efficient strategy to reduce rotavirus-associated morbidity and mortality. Compared with classical virus attenuation strategies, reverse genetics approaches such as codon deoptimization are safer, more time-saving, more cost-effective, and more controllable. The present study describes the development of an oral live-attenuated rotavirus vaccine candidate using codon deoptimization. Based on a simian rotavirus SA11 strain, eight gene segments, encoding the structural proteins VP1, VP2, VP3, and VP6, and the non-structural proteins NSP2, NSP3, NSP4, and NSP5, were subjected to codon deoptimization. Attenuated rotavirus by multi-segment codon deoptimization (MS8cd) exhibited markedly attenuated replication both *in vitro* and *in vivo*, attributable to reduced protein production independent of mRNA stability. Despite the attenuation, MS8cd elicited robust systemic and mucosal antibody responses which were sufficient to protect neonatal mice against challenge with wild-type rotavirus in a maternal immunization model. To alter the immunogenicity, MS8cd was manipulated to encapsidate outer capsid proteins of several prevalent human rotaviruses. These reassortants exhibited altered antigenic and immunogenic properties associated with the differing genotypes of the outer capsid proteins. In conclusion, this study describes the generation of promising rotavirus vaccine candidates attenuated by codon deoptimization. They are capable of eliciting genotype-specific and broad-spectrum protective immunity against circulating strains of rotavirus. This represents a rapid-response platform for the development of novel vaccines against emerging variants.

## Author summary

Rotavirus infection causes acute diarrhea in infants and young children, resulting in underestimated pediatric public health issues, especially in low-income countries. In the absence of specific antiviral therapies, vaccination remains the most effective strategy for reducing rotavirus-associated morbidity and mortality. Because of technical limitations, reverse genetics approaches have not been utilized in the development of rotavirus vaccines. The present study found that a codon deoptimized rotavirus showed highly attenuated replication *in vivo*, suggesting the safety of this live-attenuated vaccine. In addition, this codon deoptimized rotavirus elicited robust immune responses in mouse models, protecting neonatal mice from infection with wild-type rotavirus. These findings demonstrate that the codon deoptimized rotaviruses are effective candidates for live-attenuated vaccines and underscore the potential of codon deoptimization as a rapid-response platform for novel vaccine development.

## Introduction

Group A rotaviruses, common pathogens of infectious diarrhea in humans, have an 11-segmented double-stranded RNA genome of full length ~18.6 kb. Rotavirus 11 gene segments encode six structural proteins (VP1–4, VP6, and VP7) and six non-structural proteins (NSP1–6). The spike protein VP4 and outer capsid protein VP7 exhibit high genetic diversity, with combinations of these variants generating multiple genotypes. VP4 determines the P genotypes, which range from P[1] to P[58], whereas VP7 determines the G genotypes, which range from G1 to G42 (as of September 2025) [1,2]. Typically, a rotavirus genotype is denoted by the combination of its VP7 (G) and VP4 (P) genotypes.

Rotavirus infection causes acute viral gastroenteritis in infants and young children, with clinical manifestations that include watery diarrhea, vomiting and abdominal pain [3]. Because infants and young children are more vulnerable to dehydration, rotavirus infection could be life-threatening if left untreated. Limitations in the development of antiviral drugs to treat rotavirus infection have resulted in vaccination becoming the primary and most efficient approach to reduce the incidence rate of rotavirus infection and prevent severe symptoms and death. Two oral live-attenuated rotavirus vaccines, Rotarix and RotaTeq, introduced in 2006, have significantly altered the trajectory of rotavirus infection worldwide [4]. Despite the contribution of rotavirus vaccines to reduced morbidity and mortality, rotavirus remains the leading cause of infectious diarrhea with estimated 128,500 deaths in 2016 worldwide [5]. The diversity of genotypes presents a potential challenge for the development of rotavirus vaccines designed to cover a wide range of genotypes. Moreover, some genotypes have been reported to be less susceptible to antisera derived from vaccinated individuals [6,7]. These findings indicate the need for continued efforts towards the proactive development of novel vaccines.

Because live-attenuated vaccines consist of intact viruses, the immune responses they elicit are robust, long-lasting, and antigenically broad. Oral live-attenuated rotavirus vaccines simulate the fecal-oral transmission of viruses to induce persistent local immune responses in the intestinal mucosa, such as the induction of secretory IgA, thereby effectively preventing rotavirus infection and transmission [8]. Advances in reverse genetics have led to the attenuation of viruses through approaches, such as codon deoptimization [9]. Codon deoptimization exploits the codon usage bias to replace viral codons with the most rarely used synonymous codons in human cells without affecting the amino acid sequences of the encoded proteins. Consequently, these changes affect mRNA stability, protein translation efficiency and protein folding by rare tRNA usage and altered RNA secondary structures [10–15]. Codon deoptimization has been utilized to attenuate various RNA viruses [16–19], with this method found to be safer, more time-saving and cost-effective than the classical virus attenuation strategy involving multiple passages through non-human hosts [20]. However, owing to the delayed establishment of rotavirus reverse genetics system, rotavirus attenuation via codon deoptimization has not been reported.

The present study describes the development of live-attenuated rotavirus vaccine candidates by codon deoptimization using a reverse genetics system for the simian rotavirus SA11 strain [21]. The multi-segment (8-segment) codon deoptimized rotavirus (MS8cd), with highly attenuated replication *in vitro* and *in vivo*, effectively induced systemic and mucosal antibody responses that protected neonatal mice from diarrhea in a maternal immunization model. Furthermore, the immunogenic breadth of MS8cd was enhanced by incorporating outer capsid proteins of prevalent human rotavirus strains based on the MS8cd backbone, allowing the development of multivalent vaccine candidates. These findings suggest that codon deoptimization is a promising rapid-response strategy for vaccine development and support the use of codon deoptimized rotaviruses as effective live-attenuated vaccine candidates.

## Results

### Design and generation of a panel of single-segment and multi-segment codon deoptimized rotaviruses

Eight segments of the rotavirus SA11 genome, including those encoding the structural proteins VP1, VP2, VP3, and VP6 and the non-structural proteins NSP2, NSP3, NSP4, and NSP5, all of which are indispensable for rotavirus replication, were subjected to codon deoptimization. By contrast, the NSP1 gene, which has been reported to be inessential for rotavirus replication [22], was not subjected to codon deoptimization. Moreover, the spike protein VP4 and the outer capsid protein VP7 were left unmodified, so as not to interfere with the antigenicity of recombinant viruses and to allow them to be replaced by different genotypes of the VP4 and/or VP7 genes for broad-spectrum antibody induction.

**Fig 1:**
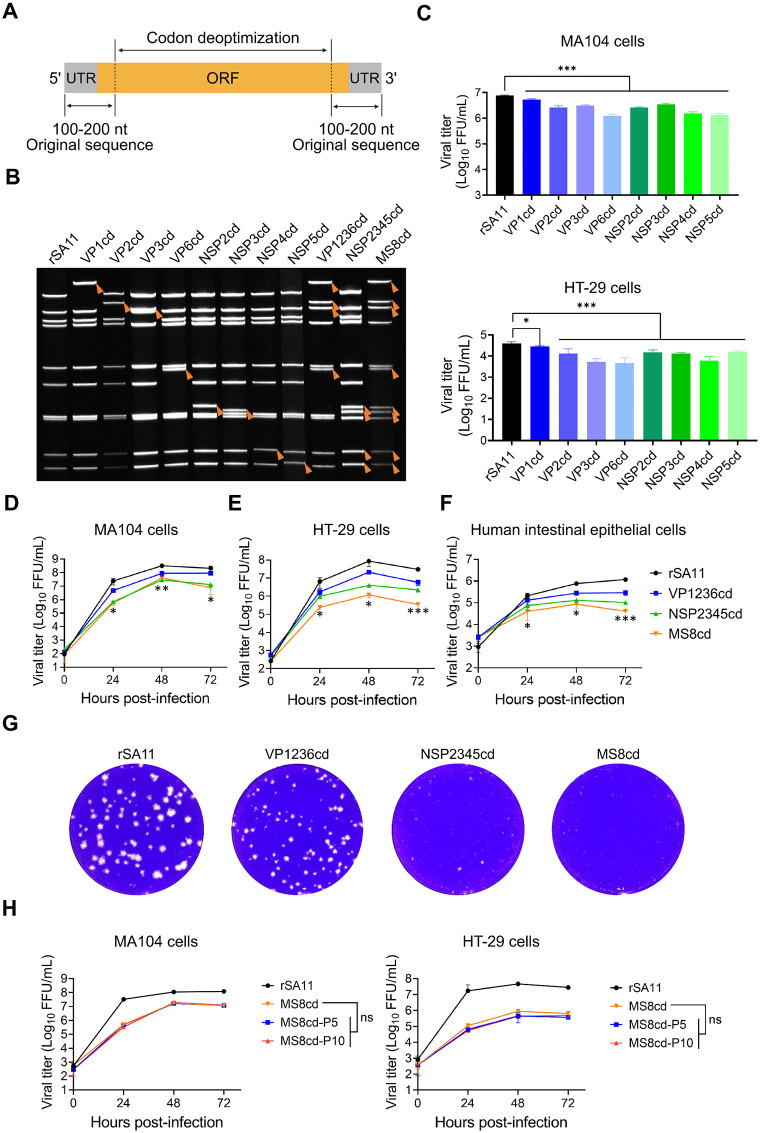
Design, generation, and *in vitro* characteristics of codon deoptimized rotaviruses. **(A)** Illustration of a rotavirus particle and codon deoptimized regions of rotavirus gene segments. **(B)** Electrophoresis of rotavirus double-stranded RNA genomes (top to bottom, VP1, VP2, VP3, VP4, NSP1, VP6, NSP2, NSP3, VP7, NSP4, and NSP5 gene segments). Viral segments with codon deoptimization migrate slower on the polyacrylamide gel electrophoresis (indicated with orange arrowheads). **(C)** Replication of single-segment codon deoptimized rotaviruses in MA104 and HT-29 cells. Cells were infected with viruses at a multiplicity of infection (MOI) of 0.01 and harvested at 24 hours post-infection (hpi). Data show the means of three biological replicates. Error bars, SD. P values calculated by one-way ANOVA (*p < 0.05, ***p < 0.001). (**D**, **E** and **F)** Multi-step viral growth kinetics of multi-segment codon deoptimized rotaviruses in **(D)** MA104 cells, **(E)** HT-29 cells, and (F) human intestinal epithelial cells. Cells were infected with viruses at an MOI of 0.01 and harvested at the indicated timepoints. Data show the means of three biological replicates. Error bars, SD. P values comparing rSA11 and MS8cd calculated by t-tests (*p < 0.05, **p < 0.01 ***p < 0.001). **(G)** Representative images of plaques formed by rSA11 and multi-segment codon deoptimized rotaviruses on MA104 cells. **(H)** Multi-step viral growth kinetics of serially passaged MS8cd in MA104 and HT-29 cells. MS8cd was passaged 10 times in MA104 cells, with MS8cd at passages 5 and 10 subjected to growth kinetics assays. Data show the means of three biological replicates. Error bars, SD. P values comparing MS8cd with MS8cd-P5 or MS8cd-P10 calculated by one-way ANOVA, with p > 0.05 considered statistically non-significant.

Packaging signals on each gene segment of segmented viruses, including rotaviruses, are considered indispensable in ensuring that each of these segments is packaged into a single viral particle during assembly [23]. Although the packaging signals of rotavirus have not yet been fully determined, alterations or deletions of the RNA sequences at the 5' and 3' terminal regions, which include the untranslated regions (UTRs) and parts of the open reading frames (ORFs), could be fatal. Therefore, in this study, each gene segment mentioned above was deoptimized, except for 100–200 base pairs at each terminal region, by replacing every viral codon with a rare human codon (Fig 1A). The nucleotide mutation rates of codon deoptimized gene segments ranged from 16.1% to 19.9%, with the GC content increasing following codon deoptimization (Table 1).

**Table 1:** Mutation rate, GC content, CAI (codon adaptation index) calculated based on *Homo sapiens*, CpG frequency in ORFs of each wild-type and codon deoptimized gene segment.

VP1 segment	Mutation rate (%)	GC content (%)	CAI	CpG frequency (%)
rSA11	—	33.7	0.63	2.8
Codon deoptimized	19.6	41.7	0.58	4.9
VP2 segment	Mutation rate (%)	GC content (%)	CAI	CpG frequency (%)
rSA11	—	32.7	0.63	2.0
Codon deoptimized	19.3	40.7	0.58	3.5
VP3 segment	Mutation rate (%)	GC content (%)	CAI	CpG frequency (%)
rSA11	—	28.6	0.65	1.6
Codon deoptimized	19.1	39.2	0.60	3.7
VP6 segment	Mutation rate (%)	GC content (%)	CAI	CpG frequency (%)
rSA11	—	38.2	0.68	2.8
Codon deoptimized	17.7	42.7	0.61	4.4
NSP2 segment	Mutation rate (%)	GC content (%)	CAI	CpG frequency (%)
rSA11	—	32.3	0.67	1.6
Codon deoptimized	19.9	40.7	0.61	3.2
NSP3 segment	Mutation rate (%)	GC content (%)	CAI	CpG frequency (%)
rSA11	—	32.5	0.67	1.5
Codon deoptimized	18.6	42.3	0.59	5.0
NSP4 segment	Mutation rate (%)	GC content (%)	CAI	CpG frequency (%)
rSA11	—	34.3	0.64	2.5
Codon deoptimized	16.1	41.1	0.62	2.1
NSP5 segment	Mutation rate (%)	GC content (%)	CAI	CpG frequency (%)
rSA11	—	36.7	0.70	2.8
Codon deoptimized	17.5	45.7	0.59	7.4

The recombinant rotaviruses carrying each codon deoptimized segment were successfully generated through a reverse genetics system for a simian rotavirus SA11 strain [21]. The generated viruses were confirmed by sequencing and dsRNA electrophoresis (Fig 1B). The altered nucleotide composition caused the codon deoptimized segments to migrate more slowly in polyacrylamide gels compared to the segments of wild-type recombinant SA11 (rSA11). The replication ability of single-segment codon deoptimized rotaviruses was tested to identify the optimal segments for rotavirus attenuation. Unexpectedly, single-segment codon deoptimization resulted in a statistically significant but modest (< 10-fold) reduction in viral replication compared to that of rSA11 in both MA104 and HT-29 cells (Fig 1C). This reduction suggested that single-segment codon deoptimization alone was insufficient to substantially attenuate rotavirus replication.

### *In vitro* characteristics of multi-segment codon deoptimized rotaviruses

To efficiently attenuate rotavirus, we generated multi-segment codon deoptimized rotaviruses, namely VP1236 cd, NSP2345 cd, and MS8cd, with codon deoptimization into gene segments encoding four structural proteins (VP1, VP2, VP3, and VP6), four non-structural proteins (NSP2, NSP3, NSP4, and NSP5), and all eight of the above proteins, respectively (Table 2). Replication of these multi-segment codon deoptimized rotaviruses was markedly attenuated in cultured cells, especially HT-29 cells (Fig 1D and 1E). Consistently, replication of these multi-segment codon deoptimized rotaviruses was markedly attenuated in human intestinal epithelial cells (hIECs) differentiated from human-derived induced pluripotent stem cells (iPSCs) (Fig 1F). Moreover, these multi-segment codon deoptimized rotaviruses showed markedly impaired plaque formation ability (Fig 1G), suggesting that the cytopathogenicity and ability to spread to adjacent cells were lower for these attenuated viruses than for wild-type virus. Recombinant rotavirus with the most highly attenuated replication, MS8cd, was subjected to subsequent analyses.

**Table 2:** Recombinant rotaviruses comprising different codon deoptimized gene segments.

Virus	Codon deoptimized segments
VP1236cd	VP1, VP2, VP3, VP6
NSP2345cd	NSP2, NSP3, NSP4, NSP5
MS8cd	VP1, VP2, VP3, VP6, NSP2, NSP3, NSP4, NSP5

The genetic stability of MS8cd virus was assessed by serial passage in MA104 cells. MS8cd viruses after five and ten passages (designated MS8cd-P5 and MS8cd-P10, respectively) showed a similar replication phenotype to that of the parental MS8cd virus (Fig 1H). Next-generation sequencing of viral genomes showed no revertant mutations. In addition to confirming the absence of revertant mutations in the engineered codon deoptimized regions, our analysis did not detect any evidence of recombination events or microinsertions/deletions across the genome (S1 Data). Several dominant single nucleotide variants (SNVs) were observed in the VP4 and NSP5 genes (Table 3). Because the VP4 segment was not codon deoptimized and the SNV on NSP5 appeared at the regions without codon deoptimization, these mutations were considered stochastic rather than reversions. These data suggest that the codon deoptimized rotavirus is genetically and phenotypically stable for at least ten passages through MA104 cells.

**Table 3:** Next-generation sequencing results, showing dominant mutations (frequency > 50%) in MS8cd passaged 10 times.

Gene	Nucleotide position	Type	Reference	Allele	Amino acid mutation	Frequency
VP4	1022	SNV	T	C	V338A	99.6%
NSP5	45	SNV	G	A	synonymous	85.3%
VP4	30	SNV	A	C	R7S	51.8%

### Decreased protein production contributes to rotavirus attenuation, independent of mRNA stability

**Fig 2:**
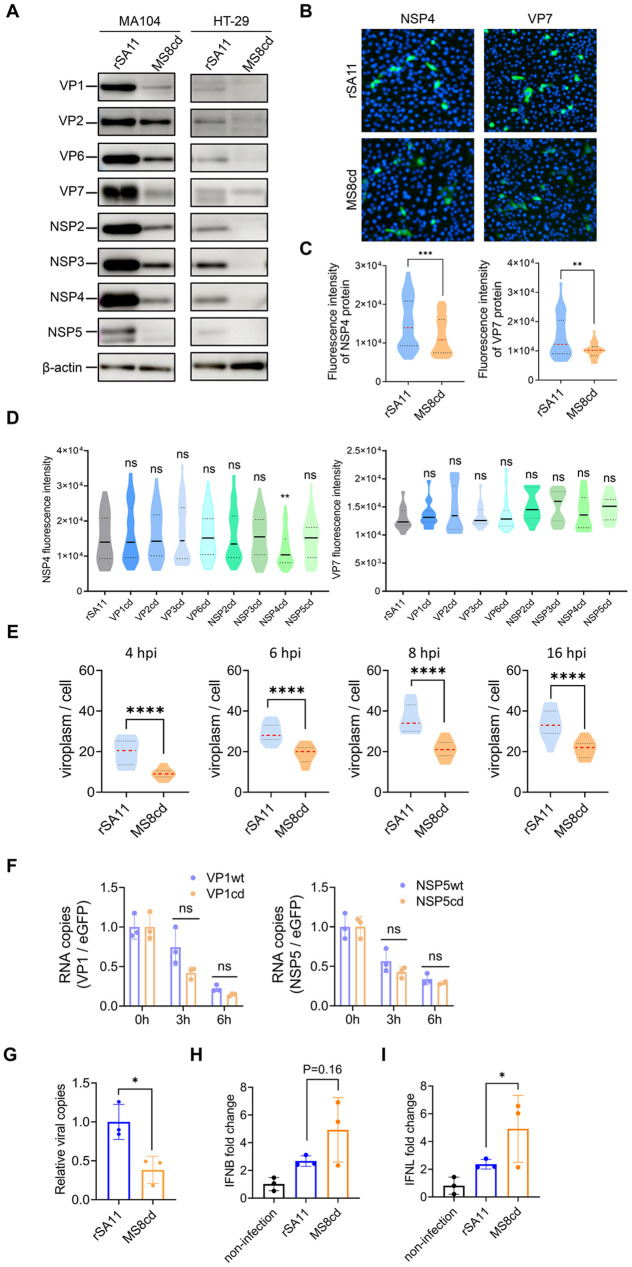
Codon deoptimization interferes with rotavirus protein production, independent of mRNA stability. **(A)** Western blotting of rSA11 and MS8cd protein production in MA104 and HT-29 cells. Cells were infected with viruses at an MOI of 1 and harvested at 8 hpi. WT: rSA11, CD: MS8cd. **(B)** Immunofluorescence images of cells infected with rSA11 and MS8cd stained with anti-NSP4 or anti-VP7 antibody at 16 hpi. **(C)** Violin plots showing the quantification of NSP4 and VP7 fluorescence intensity of single cells. All infected cells in each well of 96-well plate were counted for this analysis. The number of infected cells in each well of a 96-well plate was counted. The specific cell numbers were as follows: 77 rSA11-infected cells and 70 MS8cd-infected cells were counted for the NSP4 immunofluorecence intensity analysis; and 43 rSA11-infected cells and 42-MS8cd-infected cells were counted for the VP7 immunofluorescence intensity analysis. Violin plots showing the quantification of fluorescence intensity of single cells in **(B)**. P values calculated by one-way ANOVA (**p < 0.01, P > 0.05 was considered statistically non-significant). **(D)** Violin plots showing the quantification of NSP4 and VP7 fluorescence intensity of single cells. All infected cells in each well of each 96-well plate were counted for this analysis. p-values were calculated by one-way ANOVA (**p < 0.01; p > 0.05 was considered statistically non-significant). **(E)** Violin plots showing the amount of viroplasm formed at 4, 6, 8, and 16 hours post-infection. p-values were calculated by t-tests (****p < 0.0001). **(F)** Stability of rotavirus mRNA. HEK293T cells were transfected with dual expression plasmids encoding eGFP and rotavirus genes (VP1wt and NSP5wt) or codon deoptimized rotavirus genes (VP1cd and NSP5cd). Flavopiridol was added 24 hours after transfection. RNA was extracted 0, 3, and 6 hours after flavopiridol addition (shown on x-axis) and quantified by qRT-PCR. The mean value of wild-type gene copy numbers at 0 hour was set as a baseline, and the other copy numbers are shown relative to this baseline. Data show the means of three biological replicates. Error bars, SD. P values were calculated by t-tests, with p > 0.05 considered statistically non-significant. (**G**, **H** and **I)** Viral copies **(G)**, induction of IFN-β (**H**) and IFN-λ **(I)**. HT-29 cells were infected with rSA11 or MS8cd at an MOI of 1.0 for 24 hours. The RNA was purified and quantified by qRT-PCR. p-values were calculated by (**G**) t-test or (**H** and **I**) one-way ANOVA (*p < 0.05; p > 0.05 was considered statistically non-significant).

To gain a deeper understanding of rotavirus attenuation, the mechanistic effects of codon deoptimization were analyzed. The efficiency of viral protein translation was assessed by determining viral protein production. Western blotting showed that viral protein production was reduced in MS8cd virus (Fig 2A). Immunofluorescence assays showed that codon deoptimization resulted in a significant reduction in viral protein production at single-cell level (Fig 2B and 2C), with deoptimization affecting not only proteins encoded by deoptimized segments but also proteins encoded by non-deoptimized segments.

To further assess the effects of codon deoptimization on viral protein expression, we analyzed the levels of NSP4 (deoptimized) and VP7 (non-deoptimized) in each single-segment deoptimized virus. The results showed that codon deoptimization of a single segment (NSP4) reduced the expression of the corresponding viral protein, whereas the expression of VP7 (a non-deoptimized segment) was unaffected (Fig 2D). This indicated that codon deoptimization in our model reduced the viral protein expression levels. Additionally, these findings suggested that the attenuation observed in viruses with four or eight codon deoptimized segments may have resulted from a cumulative reduction in viral protein expression.

Viroplasm, a rotavirus inclusion body consisting of the proteins NSP2 and NSP5, has been reported to support viral replication in the cytoplasm. Viroplasms were indicated by well-colocalized NSP2 and NSP5 proteins under confocal microscopy (S1 Fig). Compared to rSA11, MS8cd virus significantly reduced the amount of viroplasm formed in single cells from 4 to 16 hours post-infection (Fig 2E). These findings suggested that codon deoptimization of eight segments interferes with protein production and impairs viroplasm formation, thereby affecting viral replication.

Given that protein production is impaired, the upstream process of protein production, the mRNA stability may also contribute to viral attenuation. Although codon deoptimization did not alter the amino acid sequences of viral proteins, it may alter the secondary structure and destabilize viral mRNA, hindering the efficiency of translation [24]. To examine the mRNA stability, we first adopted a virus-free assay using dual expression plasmids encoding a viral protein and eGFP (control of transfection efficiency) driven by independent promoters (S2 Fig). The plasmids were transiently transfected into HEK293T cells. The stability of viral mRNA was evaluated by measuring the decay of viral mRNA at different time points after flavopiridol treatment which leads to the loss of new mRNA production. Because mRNA length may affect its stability, VP1 and NSP5, the longest and shortest genes of rotavirus, respectively, were analyzed. The copy numbers of both wild-type and codon deoptimized genes were normalized to eGFP copy numbers to account for variations in transfection efficiency. However, mRNA decay did not differ significantly between wild-type and codon deoptimized rotavirus mRNAs (Fig 2F), suggesting that codon deoptimization attenuates rotavirus replication by impairing rotavirus protein production without affecting viral mRNA stability.

However, plasmid-driven expression may not fully recapitulate the transcriptional dynamics, RNA folding, and viroplasm environments that occur during viral infection. Therefore, we examined mRNA stability in an infection model by transfection of in vitro transcribed RNA generated via T7 RNA polymerase into rotavirus-infected cells (S3 Fig). As expected, there was no significant difference between the stabilities of the wild-type and codon deoptimized mRNAs.

Furthermore, we performed a time-course infection experiment and quantified the mRNA levels of a non-deoptimized segment (NSP1) and a deoptimized segment (VP1) to compare their relative abundances during viral infection. Both NSP1 and VP1 RNA levels were reduced in MS8cd-infected cells compared to those in rSA11-infected cells (S4 Fig), consistent with overall impaired viral replication (Fig 1D, 1E, and 1F). To assess the effect of codon deoptimization on the deoptimized segment, VP1 RNA copies were normalized to NSP1 RNA copies. No significant difference in normalized VP1 abundance was observed between rSA11- and MS8cd-infected cells (S4 Fig), suggesting that codon deoptimization did not substantially affect mRNA stability. Taken together, these data suggested that viral attenuation by codon deoptimization was more likely attributable to impaired translation than to reduced mRNA stability.

To investigate the mechanisms underlying viral attenuation, we examined the effects of an increased GC content. The enriched GC content can be recognized by the innate immune system during viral infection. As codon deoptimization was accompanied by an increase in the GC content in the recoded segments, we assessed IFN-β and IFN-λ induction in HT-29 cells. Despite reduced replication (Fig 2G), MS8cd induced higher levels of IFN-β and IFN-λ compared to those induced by rSA11 (Fig 2H and 2I). These results suggested that an increased GC content may enhance the recognition of MS8cd by innate immune pathways, thereby contributing to the attenuation of viral replication.

To distinguish between the effects of increased GC content and codon deoptimization, recombinant viruses carrying each GC-enriched segment without the introduction of human rare codons were also generated (VP1high, VP2high, VP3high, VP6high, NSP2high, NSP3high, NSP4high, and NSP5high). GC content and codon adaptation index (CAI) are summarized in S1 Table. The results revealed a distinct pattern of viral replication between the codon deoptimized and GC-enriched viruses. An increased GC content exerted segment-dependent effects on rotavirus replication. The introduction of GC-enriched sequences into certain segments such as VP3 and NSP3 resulted in a mild decrease in viral replication. In contrast, the introduction of GC-enriched sequences into segments, such as VP2 and NSP5, markedly impaired replication (S5 Fig). Taken together, these results indicated that an altered GC content can negatively affect rotavirus replication and may have partially contributed to the viral attenuation observed in codon deoptimized viruses.

### Pathogenicity and replication of MS8 cd in mouse models

**Fig 3:**
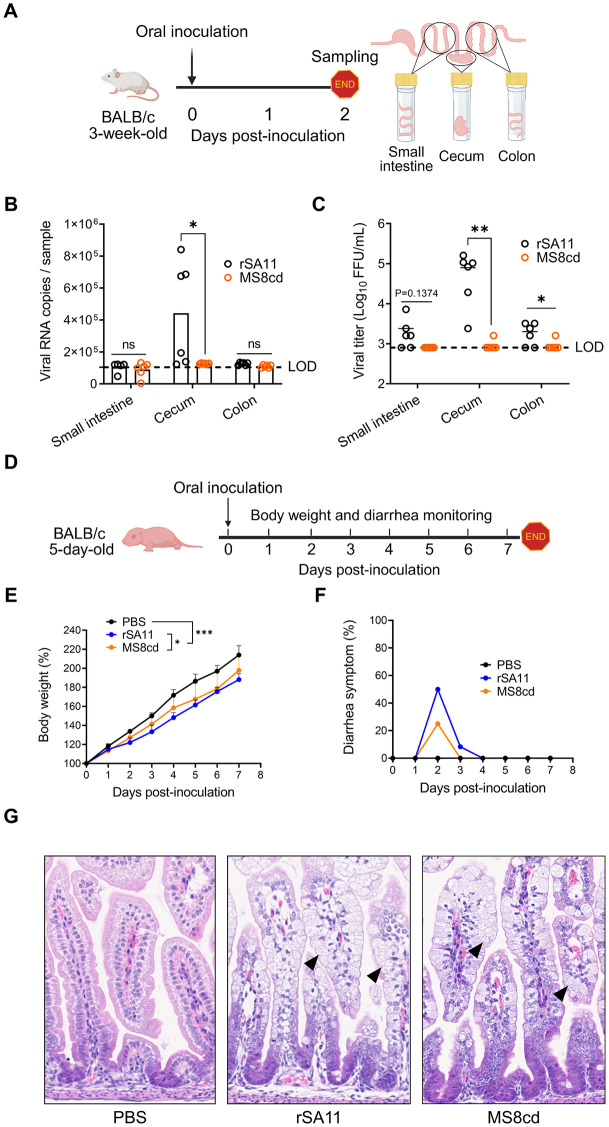
In vivo replication and pathogenicity of codon deoptimized rotavirus. **(A)** *In vivo* rotavirus replication in BALB/c mice. Each 3-week-old female mouse was orally inoculated with rSA11 or MS8cd at 1.0 × 10^7^ FFU, and samll intestine, cecum and codon samples were collected 2 days later. Created in BioRender. Chen, Z. (2026) https://BioRender.com/fr8p7v5. **(B)** Viral RNA copies in intestine, cecum and colon samples detected by qRT-PCR (n = 6 each). *p < 0.05, **p < 0.01, by Mann-Whitney U-tests, with p > 0.05 considered statistically non-significant. **(C)** Infectious viral titers in intestine, cecum and colon samples detected by immunofluorescence assays (n = 6 each). P values calculated by t-tests (*p < 0.05, **p < 0.01; p > 0.05 was considered statistically non-significant). **(D)** Timeline of the mouse challenge experiment. Each 5-day-old mouse was orally inoculated with rSA11 or MS8cd at 1.0 × 10^6^ FFU or PBS as a control, and their body weight and diarrhea symptoms were continuously monitored for one week. **(E)** Percentage of body weight change (b) relative to baseline 0 days post-inoculation (n = 6 each). P values calculated by two-way ANOVA (*p < 0.05, ***p < 0.001; p > 0.05 was considered statistically non-significant). **(F)** Diarrheal symptoms recorded for 7 days after inoculation. Vertical axis shows the percentage of mice showing diarrheal symptoms, with differences calculated by two-way ANOVA. **(G)** H&E-stained images of mouse small intestinal epithelium. Black arrows indicate the vacuolated cells.

To evaluate the *in vivo* replication of MS8cd, 3-week-old female BALB/c mice were orally inoculated with rSA11 or MS8cd. Two days post-inoculation, the mice were euthanized, and small intestine, cecum and colon samples were collected (Fig 3A). Viral RNA was extracted from tissue samples and detected by qRT-PCR. Wild-type rSA11 showed the highest viral copy numbers in the cecum samples, with levels significantly higher than those of MS8cd (Fig 3B). The infectious viral particles were trypsin-activated and detected by immunofluorescence (Fig 3C). rSA11 was detected in all three tissue samples, with the highest viral load detected in the cecum samples. The number of infectious viral particles in almost all MS8cd-infected mouse small intestine, cecum and colon samples, were under the limit of detection and significantly lower than the numbers in rSA11-infected mice, suggesting that *in vivo* viral replication was attenuated by codon deoptimization.

Pathogenicity of MS8cd was assessed using a neonatal mouse model. Five-day-old BALB/c neonatal mice were orally inoculated with rSA11 or MS8cd, and their body weight and diarrheal symptoms were monitored for 7 days (Fig 3D). Although growth retardation was milder in mice inoculated with MS8cd than in mice inoculated with wild-type virus (Fig 3E), diarrhea was observed in both groups (Fig 3F). Histopathologically, intestinal epithelial cells undergo vacuolar degeneration during rotavirus-associated diarrhea [25]. Vacuolated cells with distinct morphology were observed in over 70% of villous epithelial cells from the jejunum to the ileum of mice infected with either rSA11 or MS8cd, consistent with the observed diarrheal symptoms (Fig 3G). A previous study reported that non-replicating rotavirus induces diarrhea in mouse models [26]. Taken together, although no pathological difference was observed in a neonatal mouse model, the codon deoptimized rotavirus exhibited minimal replication in mouse intestines.

### Codon deoptimized rotavirus elicits neutralizing antibodies efficiently and protects neonatal mice from diarrhea

**Fig 4:**
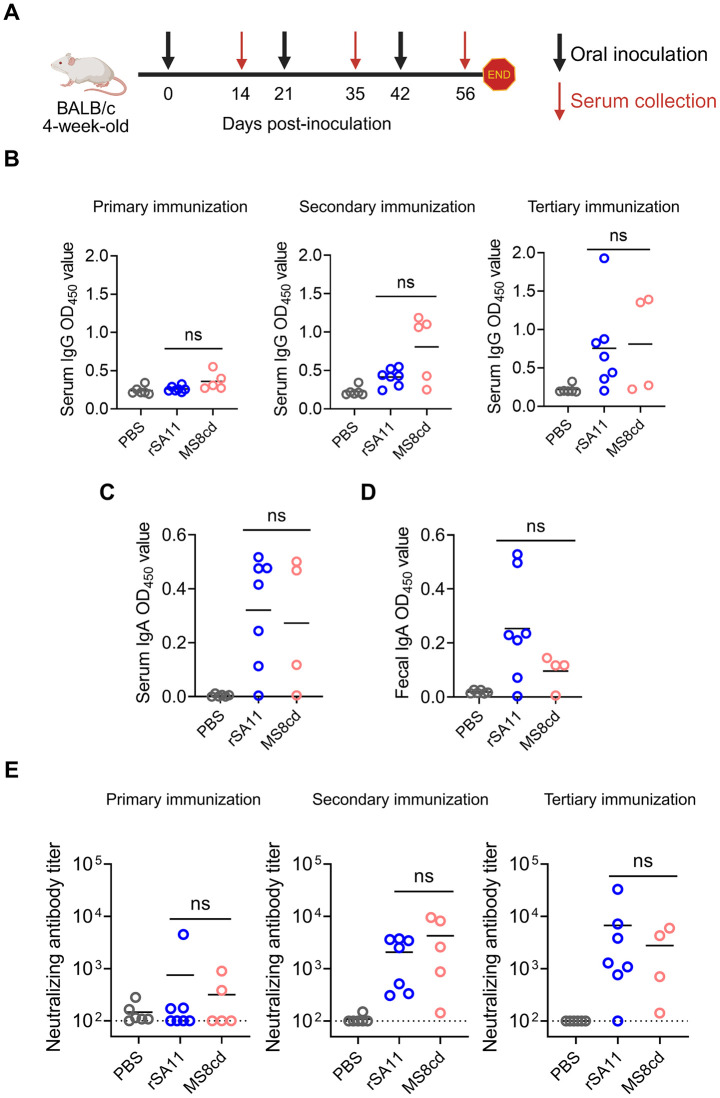
Antibody responses elicited by codon deoptimized rotavirus. **(A)** Timeline of mouse immunization. Each 4-week-old female BALB/c mice were orally inoculated with rSA11, MS8cd at 1.0 × 10^7^ FFU three times at regular intervals followed by serum collection. Created in BioRender. Chen, Z. (2026) https://BioRender.com/fr8p7v5. **(B)** Rotavirus-specific IgG in serum samples after the first, second and third inoculations, as measured by ELISA. **(C and D)** Rotavirus-specific IgA in serum (C) and fecal (D) samples following tertiary immunization, as measured by ELISA. **(E)** Rotavirus neutralizing antibody titers in serum samples following primary, secondary and tertiary immunization. P values calculated by Kruskal-Wallis tests, with p > 0.05 considered statistically non-significant.

To assess the antibody response following immunization of mice with codon deoptimized rotavirus, groups of 4-week-old female BALB/c mice were orally inoculated with rSA11, MS8cd or PBS as a negative control. The mice were immunized three times at regular intervals, with serum collected following each immunization (Fig 4A). Enzyme-linked immunosorbent assay (ELISA) for rotavirus-specific IgG (Fig 4B) and IgA (Fig 4C) in serum samples revealed that mice developed systemic antibody responses with no significant difference between the rSA11 and MS8cd groups. Mucosal antibody responses, as assessed by measuring rotavirus-specific IgA in fecal samples, did not differ significantly in the two groups (Fig 4D). Consistently, rotavirus-specific neutralizing antibodies were detected following the three immunizations, with no significant between-group differences (Fig 4E). These data indicate that codon deoptimized rotavirus and wild-type rotavirus elicit comparable antibody responses.

**Fig 5:**
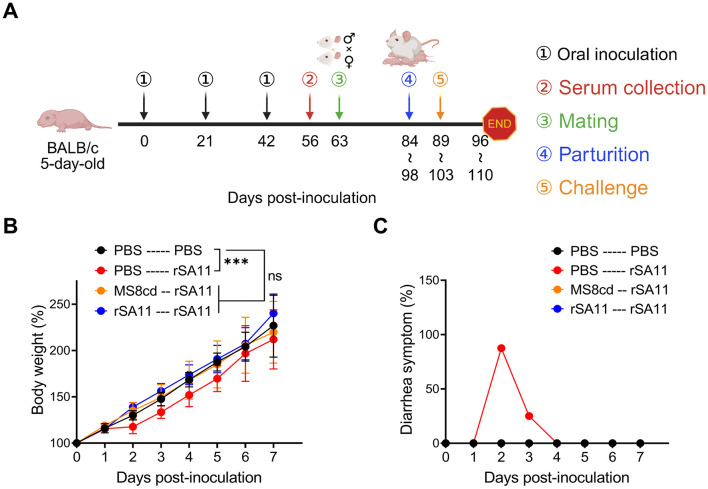
Codon deoptimized rotavirus elicits protective immunity in a maternal immunization model. **(A)** Timeline of the maternal challenge experiment. Created in BioRender. Chen, Z. (2026) https://BioRender.com/fr8p7v5. **(B)** Percentage of body weight change relative to baseline. In the graph legend, the first term indicates the inoculum used for the maternal immunization, and the second term indicates the inoculum used for the neonatal mice challenge. P values calculated by two-way ANOVA (***p < 0.001), with p > 0.05 considered statistically non-significant. **(C)** Diarrheal symptoms recorded for 7 days after inoculation. In the graph legend, the first term indicates the inoculum used for the maternal immunization, and the second term indicates the inoculum used for the neonatal mice challenge. The vertical axis shows the percentage of mice showing diarrheal symptoms.

The rotavirus SA11 strain induces diarrhea only in neonatal mice, making it difficult to perform challenge experiments after vaccination to assess the protective efficacy of the live-attenuated rotavirus vaccines. This problem was overcome by employing a maternal immunization model. At age 5 days, neonatal mice were orally inoculated with rSA11, MS8cd, or PBS. At ages 3 and 7 weeks old, only female mice were subjected for the secondary and tertiary vaccination. Following completion of the vaccination, these female mice were housed with non-littermate males. After successful mating was confirmed, the pregnant females were isolated and monitored until parturition. Five days after birth, the resulting neonatal mice were orally challenged with either rSA11 or PBS, and their body weight and diarrheal symptoms were monitored for 7 days (Fig 5A). Neonatal mice from PBS-inoculated mothers challenged with SA11 showed obvious diarrhea and significantly decreased body weight gain compared with mice without viral challenge (Fig 5B and 5C). By contrast, neonatal mice from rSA11- and MS8cd-immunized mothers challenged with rSA11 showed no diarrheal symptoms and no growth retardation as mice without viral challenge. These findings indicated that maternal immunization with codon deoptimized rotavirus conferred protection to neonatal mice against rotavirus infection.

### Generation of a panel of codon deoptimized rotaviruses with prevalent human rotavirus genotypes

**Fig 6:**
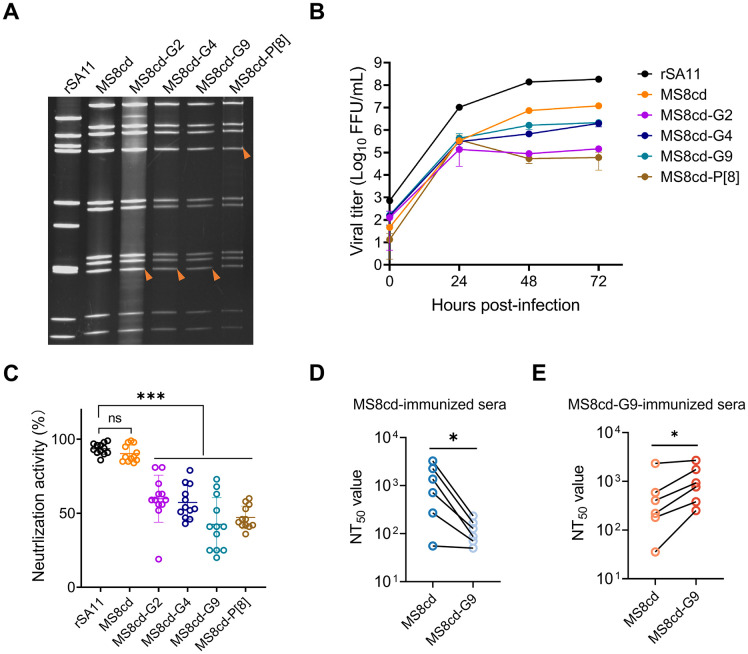
Generation of a panel of codon deoptimization attenuated rotaviruses carrying different VP4 and VP7 genes of human rotaviruses as vaccine candidates. **(A)** Electrophoresis of rotavirus double-stranded RNA of MS8cd monoreassortants carrying VP4 and/or VP7 genes of human rotaviruses. The reassorted gene segments are indicated with orange arrowheads. **(B)** Multi-step viral growth kinetics of MS8cd monoreassortants in MA104 cells. Cells were infected at an MOI of 0.01 and harvested at each indicated timepoint. **(C)** Neutralization activity of MS8cd monoreassortants carrying different VP4 or VP7 genotypes in SA11-immunized mouse serum samples. P values were calculated by one-way ANOVA (***p < 0.001), with p > 0.05 considered statistically non-significant. **(D)** Neutralizing titers of serum samples from MS8cd-immunized mice against MS8cd (G3P[2]) and MS8cd-G9 (G9P[2]). P values calculated using Wilcoxon tests (*p < 0.05). **(E)** Neutralizing titers of serum samples from MS8cd-G9-immunized mice against MS8cd (G3P[2]) and MS8cd-G9 (G9P[2]). P values calculated using Wilcoxon tests (*p < 0.05).

The *genus* rotavirus A is classified based on the genes encoding the spike protein VP4 and the outer capsid protein VP7, which define the P and G genotypes, respectively. G1P[8], G2P[8], G3P[8], G4P[8], G9P[8], and G12P[8] are prevalent genotypes in humans and present in more than 90% of circulating strains worldwide [27]. VP4 and VP7 are primary determinants for defining the antigenicity and immunogenicity of rotaviruses. Utilizing reverse genetics, we generated a panel of attenuated rotaviruses based on the codon-deoptimized MS8cd (G3P[2]) backbone, each carrying human rotavirus spike proteins or outer capsid proteins of different genotypes (Fig 6A), aiming for a wide coverage of prevalent strains.

Monoreassortant viruses displayed similar or slightly lower *in vitro* replication (Fig 6B). Attenuated rotaviruses carrying different outer capsid proteins were less susceptible to antisera derived from SA11-immunized mice (Fig 6C). These data are consistent with a previous study [28], suggesting that the antigenicity of attenuated rotavirus could be altered by switching the genotypes of the outer capsid proteins VP4 and VP7. To further assess immunogenicity, mice were immunized with either MS8cd (G3P[2]) or MS8cd-G9 (G9P[2]). Serum samples from MS8cd-immunized mice exhibited stronger neutralizing activity against the homologous MS8cd virus compared with the heterologous MS8cd-G9 (Fig 6D). Conversely, serum samples from MS8cd-G9-immunized mice neutralized MS8cd-G9 more effectively than MS8cd (Fig 6E). These data suggest that the monoreassortants can elicit genotype-specific antibody responses. Collectively, these findings indicate that the MS8cd virus could be a potent backbone for the generation of monoreassortant viruses comprising different genotypes of VP4 and VP7, providing broad-spectrum protection against prevalent and emerging rotavirus strains.

## Discussion

Rotavirus-associated morbidity and mortality have declined globally since the introduction of the first two oral live-attenuated rotavirus vaccines. Despite the success of existing vaccines, rotavirus remains the leading cause of acute gastroenteritis in infants and young children. Although it remains controversial, the emergence of variants with reduced susceptibility to existing vaccines has further complicated the spectra of rotavirus genotypes. The present study describes the generation of a panel of rotavirus live-attenuated vaccine candidates using codon deoptimization strategy. MS8cd, consisting of eight codon deoptimized gene segments, displayed attenuated replication in cell culture and mouse models. Following oral immunization, MS8cd elicited systemic and mucosal antibody responses efficiently in mouse models. A maternal immunization model found that mice vaccinated with MS8cd developed protective immunity, fully preventing the resulting neonates from growth retardation and rotavirus-associated diarrhea. Utilizing a robust reverse genetics system for simian rotavirus strain SA11, MS8cd can be used as a vaccine backbone to provide broad-spectrum protection by switching the outer capsid proteins.

Codon deoptimization is a safe and efficient approach for the attenuation of RNA viruses [16–19], however, it remains poorly understood in double-stranded RNA viruses. We initially considered that the effect of codon deoptimization might depend on the function and expression level of the target viral protein. Based on this hypothesis, we generated recombinant viruses with codon deoptimization in individual ORFs, expecting the recoding of functionally critical genes, such as NSP2, NSP5, or the polymerase VP1, to result in stronger attenuation. However, our experimental data did not support this model, as we did not observe a clear relationship between the extent of attenuation and function of the encoded protein. Consistent with this observation that codon deoptimization of a single ORF resulted in only moderate (< 10-fold) attenuation, rotavirus appears to exhibit a codon usage pattern that is relatively divergent from that of its human host compared to that of other RNA viruses [29], as reflected by its lower CAI values. This suggests that baseline codon usage is already suboptimal for the human host, which may partially limit the additional impact achievable by further codon deoptimization at a single-segment level. In line with this, a study on mammalian orthoreovirus, a 10-segmented double-stranded RNA virus related to rotavirus, showed that extensive synonymous mutations across the entire ORF of the S1 segment did not impair viral replication [30]. However, our results demonstrated that combinatorial codon deoptimization across multiple segments led to substantially enhanced attenuation. Therefore, further studies are required to clarify whether the attenuation observed in multi-segment codon deoptimization arises from the cumulative effects of modest attenuation in individual segments, or whether intersegmental RNA interactions play a more significant role in regulating viral replication [31].

In this study, codon deoptimization was accompanied by a moderate increase in GC content, making it difficult to completely separate the effects of codon usage from those of nucleotide composition. To further explore this relationship, we generated GC-enriched mutants, and found that increased GC content alone was sufficient to impair viral replication, in some cases (VP2high and NSP5high), to a greater extent than codon deoptimization. These findings suggested that changes in GC content may, in part, contribute to the attenuation observed in codon deoptimized viruses. Notably, it is also likely that these two strategies affect viral replication through partially different mechanisms, because the GC-enriched mutants exhibited higher CAI values, indicating that their reduced replication cannot be attributed to decreased codon adaptation. This observation suggested that GC-associated factors, such as RNA secondary structures, may play an important role in modulating viral replication. Future studies are required to disentangle these effects, for example, by generating recoded mutants in which GC content and codon adaptation are independently controlled, thereby enabling a more precise evaluation of their respective contributions. In the present GC-enriched mutants, CpG frequencies were also increased, which may have contributed to viral attenuation and innate immune activation. Future studies using GC- enriched mutants designed to maintain CpG frequencies comparable to those of the wild-type sequence would help distinguish the effects of increased GC content from those of CpG enrichment.

As SA11 is not a natural murine virus, viral replication in mice was limited even at high inoculation doses (Fig 3B and 3C). Therefore, the *in vivo* results should be interpreted within the constraints of this model. Although MS8cd attenuated *in vivo* replication relative to wild-type rotavirus, it did not significantly affect pathogenicity in mouse models. Diarrhea is the most common symptom of acute infectious gastroenteritis, leading to dehydration, absorptive malnutrition, and death. Rotavirus NSP4 protein has been reported to be a viroporin, which disturbs calcium homeostasis of intestinal epithelial cells and induces secretory diarrhea. Elevated cytoplasmic Ca^2+^ is crucial to rotavirus replication and virion assembly, with rotavirus-associated diarrhea being dependent on viral replication [32]. Ultraviolet-inactivated, non-transcriptional rotavirus and single-round infectious rotavirus, however, have been found to induce diarrhea in mouse models [33,34]. These findings suggest that replication-independent pathways contribute to rotavirus pathogenesis [35]. Diarrhea induced by replication-defective rotavirus may be an active host defense strategy to eliminate the pathogen through cytoplasmic dsRNA sensing and type Ⅲ interferon (IFN-λ) pathways [36]. Thus, MS8cd may induce diarrhea despite its minimal replication in mouse intestines, likely through activation of innate immune sensing pathway of the host rather than the replication-dependent mechanisms. Regarding this limitation, it is unlikely for us to perform mouse challenge experiment to confirm the virulence of the serially passaged MS8cd. Therefore, the evaluation of alternative animal models such as non-human primates may provide a more physiologically relevant assessment of vaccine safety and translational potential in future studies. Further advances in murine rotavirus reverse genetics are required to adapt this approach to a murine rotavirus strain for a more translative understanding of its immunoprotective potential in a natural host.

In our mouse model, replication of MS8cd in the mouse intestine was minimal and below the limit of detection (Fig 3B and 3C). However, the MS8cd strain in this study was able to efficiently elicit systemic and mucosal antibody responses (Fig 4B–4E). In contrast, previous studies have shown that inactivated rotaviruses lose the ability to induce neutralizing antibody responses [37]. These findings suggested that MS8cd retains a certain level of replication or antigen expression in the intestine that is sufficient to stimulate adaptive immune responses. This highlights the potential distinction between codon deoptimized and inactivated viruses, although further studies are required to directly assess viral replication and immunogenicity in more physiologically relevant models.

Employing a maternal immunization model, we observed that mice can develop protective immunity by MS8cd vaccination. In contrast to humans, transplacental transfer of antibodies is limited in mice [38]. Rather, maternal antibodies, particularly IgGs and secretory IgAs, are mainly transferred postnatally through breast milk, providing the neonates with passive immunity against pathogens [38]. During breastfeeding, maternal IgGs are absorbed to the bloodstream through active transport via the neonatal Fc receptor (FcRn) expressed on the intestinal epithelial cells of neonates, whereas IgAs remain in the gut, providing mucosal immunity [39]. Similar maternal vaccination strategies have been successfully applied to livestock to confer passive protection against enteric pathogens, supporting the biological relevance of this approach. In the maternal immunization model, maternal antibodies induced by rSA11 and MS8cd may have been transferred to the gut of neonatal mice, neutralizing orally inoculated rotavirus, protecting these neonatal mice from diarrhea and growth retardation. These findings suggest that MS8cd is highly immunogenic and has the potential to be a novel live-attenuated vaccine.

Use of a reverse genetics approach enabled the generation of a panel of monoreassortant viruses carrying different genotypes of outer capsid proteins on an attenuated MS8cd backbone. These attenuated rotaviruses with altered antigenicity may elicit broad-spectrum protection against prevalent strains and emerging variants. Despite the use of a robust reverse genetics system for the SA11 strain, generating recombinant viruses with simultaneous reassortment of the VP4 and VP7 genes based on the attenuated backbone remains difficult. Co-reassortment of the VP4 and VP7 genes was found to substantially impair viral replication when introduced into a rSA11 backbone [28]. Improving the efficiency and flexibility of reverse genetics systems is critical to broadening antigenic coverage and enhance immunogenicity. A major challenge in the development of live-attenuated vaccines lies in achieving an optimal balance between viral replication and pathogenicity. Deeper insights into virological mechanisms of rotavirus could inform future strategies for engineering viral genomes. In addition, although rotavirus vaccine escape due to strain variation is not currently considered a major clinical concern, codon deoptimization provides a conceptual platform for the rational attenuation of rotaviruses and may offer an additional strategy for vaccine design in the future.

In conclusion, the present study described the development of oral live-attenuated rotavirus vaccine candidates via codon deoptimization using an optimized reverse genetics system for the rotavirus SA11 strain. Our findings provide insight into the sequence determinants of viral replication and attenuation, highlighting the contributions of codon usage and nucleotide composition. In this regard, codon deoptimization may serve as a useful tool for rational vaccine design and for generating attenuated viral strains with defined genetic properties.

## Materials and methods

### Ethics statement

All animal experiments were approved by the institutional animal ethics committee (Approval number: BidouR03-10–0).

### Plasmids

Virus rescue plasmids encoding each rotavirus gene segment flanked by a T7 promoter and hepatitis D virus (HDV) ribozyme (named pT7-VP1SA11, pT7-VP2SA11, pT7-VP3SA11, pT7-VP4SA11, pT7-VP6SA11, pT7-VP7SA11, pT7-NSP1SA11, pT7-NSP2SA11, pT7-NSP3SA11, pT7-NSP4SA11, and pT7-NSP5SA11) were used to generate recombinant viruses. Also utilized were pCAG vector plasmids containing rotavirus NSP2 (pCAG-NSP2SA11) or NSP5 (pCAG-NSP5SA11) or vaccinia virus D1R (pCAG-D1R) or D12L (pCAG-D1L) [21]. Virus rescue plasmids encoding codon deoptimized viral genes (named pT7-VP1cd, pT7-VP2cd, pT7-VP3cd, pT7-VP6cd, pT7-NSP2cd, pT7-NSP3cd, pT7-NSP4cd, and pT7-NSP5cd) and high-GC viral genes (named pT7-VP1high, pT7-VP2high, pT7-VP3high, pT7-VP6high, pT7-NSP2high, pT7-NSP3high, pT7-NSP4high, and pT7-NSP5high) were constructed similarly to the virus rescue plasmids described above. All codon deoptimized plasmids were synthesized by GenScript. The sequence data of codon-deoptimized and high-GC segments were deposited in S2 Data. CAI was calculated using Biologicscorp. All the plasmids are available with reasonable request.

### Viruses, cells, and hIECs

Viruses: The codon deoptimized rotaviruses were generated by a plasmid-based SA11 reverse genetics system, as described previously with some modifications [21]. Briefly, BHK-T7 cells were seeded into a 12-well plate (1 × 10^5^ cells/well), followed the next day by co-transfection with 11 virus rescue plasmids (pT7-VP1SA11, pT7-VP2SA11, pT7-VP3SA11, pT7-VP4SA11, pT7-VP6SA11, pT7-VP7SA11, pT7-NSP1SA11, pT7- NSP2SA11, pT7- NSP3SA11, pT7-NSP4SA11, and pT7-NSP5SA11) or the corresponding plasmids with codon deoptimized genes (pT7-VP1cd, pT7-VP2cd, pT7-VP3cd, pT7-VP6cd, pT7-NSP2 cd, pT7-NSP3cd, pT7-NSP4cd, and pT7-NSP5cd), along with the four pCAG vector plasmids (pCAG-NSP2SA11, pCAG-NSP5SA11, pCAG-D1R, and pCAG-D12L). A total of 15 plasmids (0.125 µg/well each, 1.875 µg/well in total) were transfected into the cells using TransIT-LT1 (Mirus) at a DNA:reagent ratio of 1:2 (µg:µL). After 24 hours, MA104 cells (1.5 × 10^5^ cells/well) suspended in Dulbecco’s modified Eagle’s medium (DMEM) containing 0.5 µg/mL trypsin were added to the BHK-T7 cells and co-cultured for 5 days, followed by three freeze/thaw cycles. The generated viruses were passaged in fresh MA104 cells with trypsin at a final concentration of 0.5 µg/mL, without activation by a high trypsin concentration. The passaged viruses were confirmed by IFA using an anti-NSP4 antibody (see below), with the presence of the NSP4 antigen indicating successful rescue of recombinant rotaviruses. The viruses were subsequently stored at -80°C.

Cells: BHK-T7, MA104 and HT-29 cells were cultured in DMEM (Nacalai Tesque) supplemented with 5% fetal bovine serum (FBS; Gibco) at 37°C with 5% carbon dioxide. BHK-T7 cells were treated with 1 µg/mL puromycin for antibiotic selection.

hIECs: The culture and maintenance of hIECs have been described [40]. Briefly, hIECs were differentiated from human-derived iPSCs cultured in Matrigel (Corning). The hIECs were maintained in advanced DMEM/F-12 (Thermo Fisher Scientific), supplemented with 1% penicillin/streptomycin solution (Gibco), 2 mM Glutamax (Thermo Fisher Scientific), 10 mM HEPES (Thermo Fisher Scientific), 1 × B-27 (Gibco), 50 ng/mL recombinant murine EGF (PEROTECH), 10 μM SB202190 (Sigma-Aldrich), 500 nM A83-01 (Tocris), and 25% L-WRNH [41] (supernatants of cultured L cells stably expressing mouse Wnt3a, human R-spondin 1, human Noggin, and hepatocyte growth factor, HGF) at 37°C with 5% carbon dioxide. Before viral infection, the hIECs were disassociated using TrypLE Express (Thermo Fisher Scientific) and seeded onto Matrigel-coated 96-well plates as a monolayer as described below.

### Antibodies

Rabbit anti-VP1, anti-VP2, and anti-VP3 antisera were raised against synthetic SA11 VP1 (amino acid residues 373–391), and VP2 (amino acid residues 10–28), respectively (Eurofins Genomics). Mouse monoclonal anti-VP6 antibody was purchased from SANTA CRUZ Biotechnology, rabbit anti-VP7 antibody was purchased from MyBioSource. Rabbit anti-NSP2, anti-NSP3, and anti-NSP4 antisera were raised against synthetic SA11 NSP2 (amino acid residues 299–312), NSP3 (amino acid residues 143–156) and NSP4 (amino acid residues 158–171) peptides (Eurofins Genomics). Guinea pig anti-NSP5 antiserum was raised against a synthetic SA11 NSP5 peptide spanning amino acid residues 48–66 (Eurofins Genomics). Mouse monoclonal anti-β-actin antibody was purchased from Sigma-Aldrich.

### Virus titration

MA104 cells were seeded at a density of 1.5 × 10^4^/well in 96-well plates 1 day before virus titration. Viruses were serially diluted before adding to MA104 cells, and the infected cells were incubated at 37°C for 16 hours. The supernatants were decanted, and the cells were fixed in 4% formaldehyde solution and permeabilized with 0.05% Triton X-100. Primary anti-NSP4 antibody diluted in PBS containing 2% inactivated FBS was added to the cells, followed by incubation at room temperature for 1 hour on a shaker. The cells were washed three times, followed by incubation with secondary antibody and Hoechst, each diluted in PBS containing 2% inactivated FBS, at room temperature for 1 hour on a shaker, with the cells washed three times with PBS between steps. Focus-forming units were detected using Cytation 5 (Agilent Technologies).

### Virus purification

Viruses propagated in MA104 cells were collected and centrifuged at 4800 × g for 10 minutes to remove the cell debris. The supernatants were subjected to ultracentrifugation at 100,000 × g for 90 minutes [42]. The supernatants were carefully removed, and the pellets resuspended in Tris-natrium-calcium (TNC) buffer, consisting of 0.1 M Tris-HCl (pH 8.0), 0.5 M NaCl, 0.1 M CaCl_2_, and incubated overnight at 4°C. The concentrated virus solutions were added to tubes containing 2.5 mL of 55% cesium chloride and 2 mL of 40% cesium chloride, followed by isopycnic differential ultracentrifugation at 100,000 × g for 17 hours, which separated the rotavirus double-layered and triple-layered particles into two visible bands. The double-layered and triple-layered particles were extracted separately and dialyzed using dialysis cassettes against TNC buffer at 4°C for over 24 hours, with one change to fresh TNC buffer once during dialysis. The triple-layered particles were collected and used in subsequent experiments.

### Viral genome electrophoresis

Viral dsRNAs extracted using Sepasol (Nacalai Tesque) were loaded into 10% polyacrylamide gels, followed by electrophoresis for 180 minutes (180 V, 25 mA) in Tris-borate-EDTA (TBE) buffer; consisting of 89 mM Tris, 89 mM boric acid, and 2 mM EDTA. The dsRNAs were post-stained with GelRed (Biotium) for 20 minutes before visualization.

### Viral multi-step growth kinetics

For cultured cells: MA104 cells at a density of 7.5 × 10^4^/well or HT-29 cells at a density of 2 × 10^5^/well were seeded into 24-well plates. The next day, the cells were washed once with serum-free DMEM, viruses were added, and the cells were incubated at 37°C for 1 hour. After washing once with serum-free medium, the cells were incubated at 37°C with trypsin at a final concentration of 0.5 μg/mL in serum-free medium. The cells were harvested 0, 24, 48, and 72 hours after infection and subjected to three freeze/thaw cycles before titration.

For hIECs: hIECs were subjected to differentiation as described [39]. Briefly, hIECs cultured three- dimensionally in Matrigel were rinsed with D-PBS (-), followed by the addition of TrypLE Express with 10 μM of the ROCK inhibitor Y27632 to release them from Matrigel and dissociate them into single cells. The dissociated hIECs were seeded in Matrigel-coated 96-well plates in culture medium supplemented with 10 μM ROCK inhibitor and incubated for 48 hours. The culture medium was replaced with fresh differentiation medium, consisting of advanced DMEM/F-12 supplemented with 1% penicillin/streptomycin solution, 2 mM Glutamax, 10 mM HEPES, 1 × B-27, 50 ng/mL recombinant murine EGF (PEROTECH), 500 nM A83-01 (Tocris), and 12.5% L-RN (supernatants of cultured L cells stably expressing human R-spondin1 and human Noggin). After an additional 48 hours, 6 days after seeding, viruses were added to monolayered IECs, which were incubated at 37°C for 1 hour, washed three times with prewarmed differentiation medium, and incubated at 37°C with differentiation medium (without serum) containing trypsin at a final concentration of 0.5 μg/mL. The viruses were harvested 0, 24, 48, 72 hours after infection. Following three freeze/thaw cycles, viral titers were measured by immunofluorescence assays using MA104 cells.

### Plaque assays

MA104 cells were seeded at a density of 1.5 × 10^5^/well in 12-well plates. The cells were washed once with serum-free DMEM, viruses were added, and the cells were incubated at 37°C for 1 hour. The cells were washed once with serum-free DMEM, serum-free DMEM containing 0.8% sea plaque agarose and 0.5 μg/mL trypsin was added, and the cells were incubated at room temperature for 1–2 hours until the agarose gel had completely solidified. The cells were incubated at 37°C for an additional 4–5 days, fixed in 4% formaldehyde solution and stained with crystal violet.

### Next-generation sequencing analysis of serially passaged virus

MS8cd passaged five and ten times in MA104 cells was concentrated by ultracentrifugation. The virus preparations were resuspended in PBS and treated with DNase and RNase to remove cellular DNA and RNA. Viral genomes were extracted using ISOSPIN viral RNA kits (NIPPON GENE). Viral RNA was denatured at 95°C for 90 s. cDNA synthesis was subsequently carried out using the GenNext RamDA-seq Single Cell Kit. The resulting cDNA was then utilized for library preparation with the Illumina Nextera XT DNA Library Preparation Kit, following the manufacturer’s protocol. Paired-end sequencing (101 bp) was performed on the Illumina NovaSeq 6000 platform. Raw sequencing reads were processed by adapter trimming using Trimmomatic v0.38. The trimmed reads were then subjected to de novo assembly using CLC Genomics Workbench version 24.0.2, and the genome sequence was constructed. The analyzed data was deposited in S1 Data.

### Western blotting

MA104 cells were seeded at a density of 3 × 10^5^/well and HT-29 cells were seeded at a density of 8 × 10^5^/well in 6-well plates. The cells were infected with rSA11 or MS8cd at a multiplicity of infection (MOI) of 1.0. After incubation at 37°C for 1 hour, the supernatants were removed and the cells were washed with PBS and cultured in DMEM supplemented with 5% FBS at 37°C for 8 hours. The cells were subsequently washed with PBS and lysed in RIPA buffer, consisting of 1M Tris-HCl (pH 7.4), 1M NaCl, 10% SDS, 10% Triton X-100, 10% sodium deoxycholate and protease inhibitors. The cell lysates were centrifuged at 16,400 × g for 30 minutes to remove insoluble debris, and the clarified lysates were diluted with 2x sample buffer (0.5M Tris-HCl [pH 6.8], 10% SDS, 2-mercaptoethanol, glycerol and 1% bromophenol blue) and heated at 95°C for 5 minutes for thermal denaturation. The samples were loaded onto 10% polyacrylamide gels for electrophoresis, followed by transfer to nylon membranes. Rotavirus VP1, VP2, VP6, VP7, NSP2, NSP3, NSP4, and NSP5 proteins and the cell protein β-actin as a loading control were detected using corresponding antibodies.

### Immunofluorescence assays

MA104 cells were seeded at a density of 7.5 × 10^4^/well on cover slips in 24-well plates, followed by the addition of rSA11 or MS8cd at an MOI of 0.5 and incubation at 37°C for 2, 4, 6, 8, or 16 hours. The cells were rinsed with PBS, fixed in 4% formaldehyde, permeabilized with 0.05% Triton-X100, and incubated with 5% FBS in PBS for 1 hour to block non-specific binding. Rotavirus NSP2 was stained with anti-rabbit 488 fluorescence antibody, rotavirus NSP5 was stained with anti-guinea pig 594 fluorescence antibody and cellular nuclei were stained with Hoechst. The cells were examined using a C2 + Eclipse Ti2 confocal microscope (Nikon), with co-localized NSP2 and NSP5 proteins being indicative of rotavirus viroplasms.

### RNA stability assays

For the virus-free assay: HEK293T cells in 12-well plates were transiently transfected with dual expression plasmids encoding eGFP and viral sequences at 1 μg/well, followed by the addition 24 hours later of flavopiridol (alvocidib, Selleck) at a final concentration of 2 μM. Cellular RNA was extracted 0, 3, and 6 hours after flavopiridol addition using RNA extraction kits (ISOSPIN). The numbers of RNA copies were determined by qRT-PCR using primers and probes for VP1 (forward primer, 5’-AAAGCTGTACAATGGGGAAGT-3’; reverse primer, 5’-ACGATAACCCATTCTTTGAGTTCT-3’; probe, 5’-[FAM]-TCACAATCTGCAGTTCAAATTCC-[TAM]-3’) and NSP5 (forward primer, 5’-AGCGCTACAGTGATGTCTCTC-3’; reverse primer, 5’-TGACGTTGTTGAAGACGATTCAT-3’; probe, 5’-[FAM]-TGACGTGACGAGTCTTCCTT-[TAM]-3’) and primers for eGFP (forward primer, 5’-AGTCCGCCCTGAGCAAAGA-3’; reverse primer, 5’-TCCAGCAGGACCATGTGATC-3’).

For the RNA stability assay in an infection model: The HEK293T cells were infected with rSA11 at an MOI of 1.0 for 8 hours. The ARCA-capped RNA transcripts lacking a poly(A) tail of wild-type VP1 (VP1wt) and codon deoptimized VP1 (VP1cd) were synthesized using mMESSAGE mMACHINE T7 Ultra kit (Thermo Fisher Scientific) and transfected into infected HEK293T cells (1 μg/well in 24-well plates) using TransIT-mRNA (RNA: reagent: boost = 1:2:2). After 16 hours incubation, the mRNA was removed and cycloheximide was added as a final concentration of 125 μM to stop translation of viral RdRp and to indirectly prevent synthesis of viral mRNA. At 0 and 4 hours after cycloheximide addition, the viral RNA was collected and quantified by qRT-PCR (THUNDERBIRD Probe One-step qRT-PCR kit, TOYOBO) using primers the same as those described above. Because the qPCR primers amplify VP1 sequences present in both transfected VP1 in vitro-transcribed RNA and rSA11-derived viral RNA, rSA11-infected cells treated with TransIT-mRNA reagent alone were included as a background control. The VP1 copy number detected in this control was subtracted from the total VP1 copy number measured in cells transfected with VP1wt or VP1cd RNA transcripts. The resulting values were used as the estimated copy numbers of transfected VP1 RNA transcripts.

For the time-course of mRNA of deoptimized and non-deoptimized segments in infected cells: MA104 cells were infected with rSA11 or MS8cd at an MOI of 10.0 for 16 hours. The RNA was purified and determined by qRT-PCR (THUNDERBIRD Probe One-step qRT-PCR kit, TOYOBO) using primers and probes specific to VP1 (as described above) and NSP1 (forward primer, 5’-TTGCTGCAATGATGACGAGT-3’; reverse primer, 5’-TATCGGAAGCATTGCCTGGG-3’; probe, 5’-[FAM]-ACATCATTGTCAGCCTAACTACGTGGCA-[TAM]-3’). Ct values for VP1 and NSP1 were first converted to copy numbers using target-specific standard curves. These viral RNA copy numbers were then normalized to the copy number of a housekeeping gene. This approach allowed us to compare normalized VP1 and NSP1 RNA levels across wild-type and mutant viruses.

### IFN induction

HT-29 cells were seeded in 24-well plates and infected with rSA11 or MS8cd at an MOI of 1.0 for 24 hours. RNA was purified using Sepasol and quantified by qRT-PCR using SYBR Green one-step kit (Bio-Rad). Primers are as follows: IFNB forward primer, 5’-CATTACCTGAAGGCCAAGGA-3’; IFNB reverse primer, 5’-CAATTGTCCAGTCCCAGAGG-3’; IFNL forward primer, 5’-AACTGGGAAGGGCTGCCACATT-3’; IFNL reverse primer, 5’-GGAAGACAGGAGAGCTGCAACT-3’.

### Challenge experiments

Pregnant female BALB/c mice were allowed to acclimate and monitored until parturition. Five days after birth, the neonatal mice were divided into three groups and orally inoculated with rSA11 or MS8cd at 1.0 × 10^6^ FFU or PBS as a negative control. Body weights and diarrheal symptoms [43] were monitored continuously for 1 week.

### Histopathological analysis

Each female BALB/c mouse was inoculated with rSA11, or MS8cd at 1.0 × 10^7^ FFU or PBS as a control. Mouse intestines were collected at 2 dpi, fixed in 10% neutral buffered formalin, and processed routinely for embedding in paraffin. The 2 μm thick tissue sections were cut and stained with hematoxylin and eosin for the histopathological evaluation. Vacuolated cells were identified based on the morphological features of the histological sections. Specifically, cells with clear cytoplasmic vacuoles were distinguished from morphologically normal cells and quantified.

### Mouse immunization

Each 4-week-old female BALB/c mouse was orally inoculated three times with SA11 or MS8cd at 1.0 × 10^6^ FFU or PBS as a control, with a 3-week interval between immunizations. The serum of each mouse was collected 2 weeks after each inoculation. Subsequently, the mice were euthanized, and fecal samples were collected from their colons. Mice were immunized with MS8cd or MS8cd-G9 at 1.0 × 10^6^ FFU twice with a 3-week interval, then the serum of each mouse was collected two weeks after the secondary immunization.

### ELISAs

Infectious virions (triple-layered particles) of rSA11 were purified by CsCl density-gradient centrifugation. A 96-well flatbottomed MaxiSorp plate (Thermo Fisher Scientific) was coated overnight at 4°C with purified virus (1 × 10^6^ FFU/well) in carbonate coating buffer (15 mM Na_2_CO_3_, 7 mM NaHCO_3_, pH 9.6). The plate was washed three times with ELISA wash buffer (PBS containing 0.05% Tween), incubated with ELISA diluent buffer (PBS containing 1% bovine serum albumin and 0.05% Tween) for 1 hour at 37°C to block non-specific binding, washed, and incubated sequentially with immunized mice sera (1:1000) and HRP-conjugated anti-mouse IgG or IgA (1:1000) (Abcam) for 1 hour each at 37°C. The HRP substrate 3,3’,5,5′-tetramethylbenzidine (Sigma-Aldrich) was added for color development, and the absorbance of each well was measured at 450 nm using a Cytation 5 Cell Imaging Multimode Reader (Agilent Technologies).

### Neutralization assays

Wild-type rSA11 or NLuc-expressing SA11 [44] pre-treated with diluted mouse serum samples at 37°C for 1 hour was incubated for 16 hours with fresh MA104 cells. Neutralizing antibody titers were evaluated by immunofluorescence assays counting the numbers of foci, as described above, or by measuring NLuc activity using a NanoGlo Luciferase Assay system (Promega). Neutralization assay using MS8cd or MS8cd-G9 viruses and sera from MS8cd-immunized or MS8cd-G9-immunized mice was performed as described above.

### Challenge experiments using a maternal immunization model

Five-day-old neonatal mice were orally inoculated with 10^6^ FFU rSA11 or MS8cd, or with PBS as a negative control. Female mice (n = 9) were divided into three groups: PBS (n = 5 dams), rSA11 (n = 2 dams), and MS8cd (n = 2 dams). Dams that failed to become pregnant or that cannibalized their pups were excluded from the analysis. Female mice were subsequently inoculated at 3 and 7 weeks-of-age. Inoculated females were subsequently housed with non-littermate males. Once successful mating was confirmed, the pregnant females were isolated and monitored until parturition. Neonatal mice from PBS-inoculated dams (n = 21 pups) were assigned to two groups: A PBS control (n = 12 pups) inoculated with PBS, and a challenge group (n = 9 pups) which was challenged with 10^6^ FFU rSA11 at 5 d after birth. Neonatal mice from SA11-immunized dams (n = 12 pups) and MS8cd-immunized dams (n = 9 pups) were challenged with 10^6^ FFU of SA11 5 d after birth. Body weight and diarrheal symptoms were monitored for 1 week.

### Statistical analysis

The data were analyzed statistically using GraphPad Prism 8 (GraphPad Software, Inc.). Data are expressed as mean values ± standard deviation of at least two independent experiments (each with three technical replicates, unless stated otherwise), with p < 0.05 considered statistically significant.

## Supporting information

S1 Fig Immunofluorescence images of viroplasm. MA104 cells were infected with rSA11 or MS8cd at an MOI of 0.5 for 8 hours. The cells were rinsed with PBS, fixed in 4% formaldehyde, permeabilized with 0.05% Triton-X100, and incubated with 5% FBS in PBS for 1 hour to block non-specific binding. Rotavirus NSP2 was stained with anti-rabbit 488 fluorescence antibody, rotavirus NSP5 was stained with anti-guinea pig 594 fluorescence antibody and cellular nuclei were stained with Hoechst. The cells were examined using a C2 + Eclipse Ti2 confocal microscope (Nikon), with co-localized NSP2 and NSP5 proteins being indicative of rotavirus viroplasms.
(TIF)

S2 Fig Protein expression of dual expression plasmid. The plasmids were transfected into HEK293T cells. After 24 h of incubation, cells were fixed and stained with an anti-NSP5 antibody.
(TIF)

S3 Fig RNA stability assay in an infection model. HEK293T cells were infected with rSA11 at an MOI of 1.0 for 8 h. The capped viral transcripts of wild-type VP1 (VP1wt) and codon deoptimized VP1 (VP1cd) were transfected into infected HEK293T cells using TransIT mRNA (RNA:reagent = 1:3). After 16 h of incubation, the mRNA was removed and cycloheximide was added to stop the translation of viral RdRp and prevent the synthesis of new viral mRNA. RNA was collected at 0 and 4 h after cycloheximide addition and analyzed by RT-qPCR. p-values were calculated using two-way ANOVA (p > 0.05 was considered statistically non-significant).
(TIF)

S4 Fig Viral mRNA abundance during infection. Viral (A) VP1 and (B) NSP1 RNA copies at 16 h post-infection in MA104 cells. p-values were calculated by two-way ANOVA (***p < 0.001). (C) VP1 RNA copies normalized to NSP1 RNA copies. p-values were calculated by two-way ANOVA (p > 0.05 was considered statistically non-significant).
(TIF)

S5 Fig Replication of recombinant rotaviruses with a GC-enriched segment. To analyze the impact of GC content on viral replication, we generated recombinant rotaviruses carrying one GC-enriched segment without introducing rare human codons. MA104 cells were infected with rSA11, single-segment codon deoptimized viruses, and single-segment GC-enriched viruses at an MOI of 1.0, and incubated for 24 h. Viral titers were determined by immunofluorescence staining. p-values were calculated using one-way ANOVA (*p < 0.05, **p < 0.01, ***p < 0.001).
(TIF)

S1 Table Mutations rate, GC content, CAI (codon adaptation index) calculated based on Homo sapiens, CpG frequency in ORFs of each wild-type, codon deoptimized and GC-enriched gene segment. (DOCX)

S1 Data NGS analysis of codon deoptimized MS8 cd after 10 serial passages. (XLSX)

S2 Data Viral sequences containing codon deoptimized or GC-enriched mutations. (DOCX)

S3 Data Raw data for figure generation. (XLSX)
