# Supplementary figures and images for: A rotavirus vaccine candidate attenuated by codon deoptimization protects neonatal mice against wild-type virus infection
Source: PLoS Pathog. 2026 Jul 7;22(7):e1014292. doi: 10.1371/journal.ppat.1014292 (PMC13340783; doi:10.1371/journal.ppat.1014292)

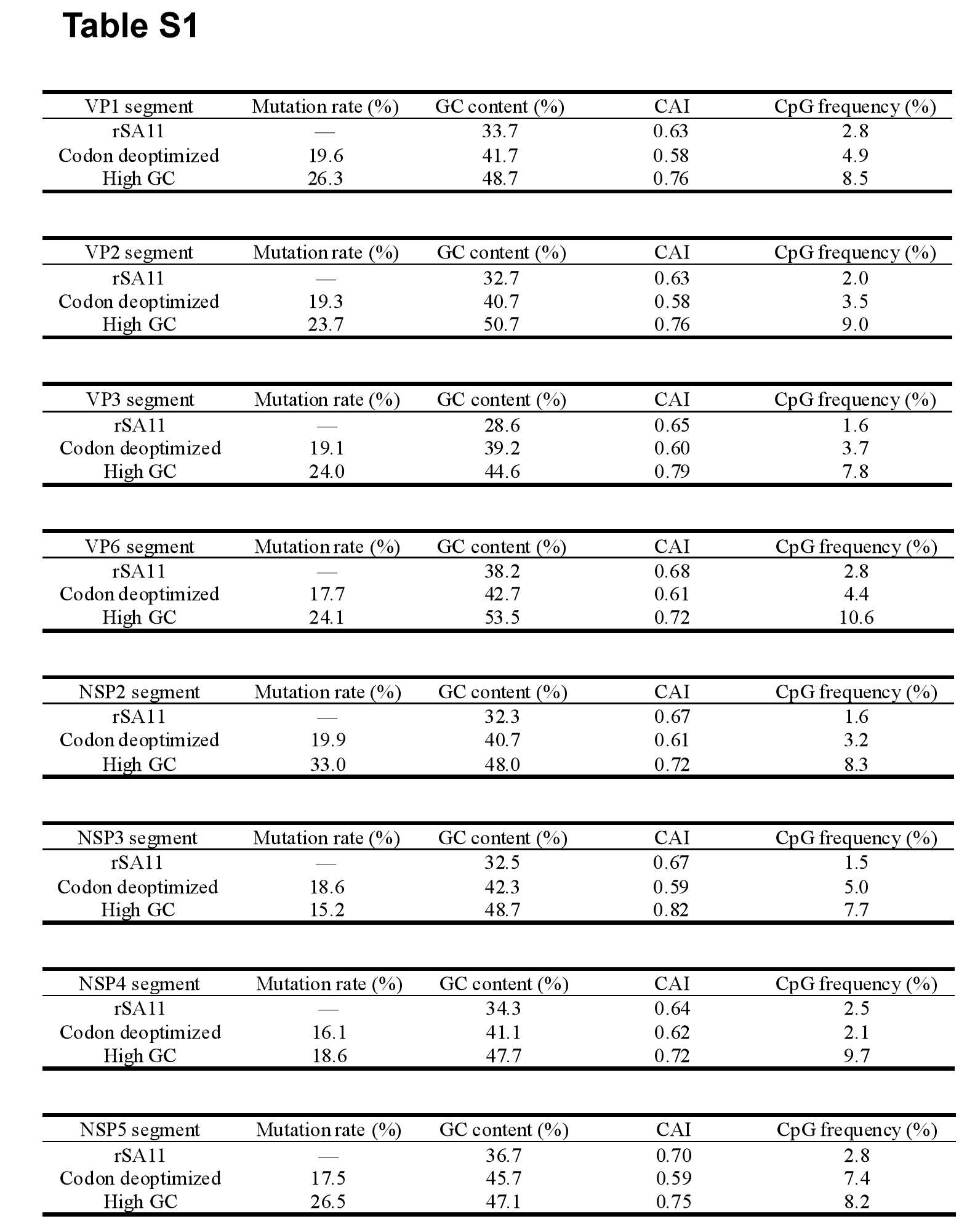

Supplement: S1 Table — (DOCX) [file ppat.1014292.s006.docx]
