# Supplementary material for: A rotavirus vaccine candidate attenuated by codon deoptimization protects neonatal mice against wild-type virus infection
Source: PLoS Pathog. 2026 Jul 7;22(7):e1014292. doi: 10.1371/journal.ppat.1014292 (PMC13340783; doi:10.1371/journal.ppat.1014292)
Supplement: S2 Data — (DOCX) [file ppat.1014292.s008.docx]

>SA11 VP1cd

ATGGGGAAGTACAATCTAATCTTGTCAGAATATCTATCATTTATATATAATTCACAATCTGCAGTTCAAATTCCAATATATTACTCTTCCAACAGTGAATTAGAAAATAGATGTATTGAATTTCATTCCAAGTGTTTAGAGAACTCAAAGAATGGGTTATCGTTAAGAAAGTTGTTTGTTGAATACAATGACGTAATAGAGAATGCTACACTACTATCGATACTATCGTACTCGTACGACAAGTACAATGCTGTAGAGAGGAAGCTAGTAAAGTACGCTAAGGGAAAGCCCCTAGAGGCTGACCTAACAGTAAATGAGCTAGACTACGAGAATAATAAGATAACATCGGAGCTATTCCCCACAGCTGAGGAGTACACAGACTCGCTAATGGACCCCGCTATACTAACATCGCTATCGTCGAATCTAAATGCTGTAATGTTCTGGCTAGAGAAGCACGAGAATGACGTAGCTGAGAAGCTAAAGGTATACAAGAGGAGGCTAGACCTATTCACAATAGTAGCTTCGACAATAAATAAGTACGGAGTACCCAGGCACAATGCTAAGTACAGGTACGAGTACGACGTAATGAAGGACAAGCCCTACTACCTAGTAACATGGGCTAATTCGTCGATAGAGATGCTAATGTCGGTATTCTCGCACGACGACTACCTAATAGCTAAGGAGCTAATAGTACTATCGTACTCGAATAGGTCGACACTAGCTAAGCTAGTATCGTCGCCCATGTCGATACTAGTAGCTCTAGTAGACATAAATGGAACATTCATAACAAATGAGGAGCTAGAGCTAGAGTTCTCGAATAAGTACGTAAGGGCTATAGTACCCGACCAAACATTCGACGAGCTAAATCAAATGCTAGACAATATGAGGAAGGCTGGACTAGTAGACATACCCAAGATGATACAAGACTGGCTAGTAGACAGGTCGATAGAGAAGTTCCCCCTAATGGCTAAGATATACTCGTGGTCGTTCCACGTAGGATTCAGGAAGCAAAAGATGCTAGACGCTGCTCTAGACCAACTAAAGACAGAGTACACAGAGAATGTAGACGACGAGATGTACAGGGAGTACACAATGCTAATAAGGGACGAGGTAGTAAAGATGCTAGAGGAGCCCGTAAAGCACGACGACCACCTACTAAGGGACTCGGAGCTAGCTGGACTACTATCGATGTCGTCGGCTTCGAATGGAGAGTCGAGGCAACTAAAGTTCGGAAGGAAGACAATATTCTCGACAAAGAAGAATATGCACGTAATGGACGACATGGCTAATGAGAGGTACACACCCGGAATAATACCCCCCGTAAATGTAGACAAGCCCATACCCCTAGGAAGGAGGGACGTACCCGGAAGGAGGACAAGGATAATATTCATACTACCCTACGAGTACTTCATAGCTCAACACGCTGTAGTAGAGAAGATGCTAATATACGCTAAGCACACAAGGGAGTACGCTGAGTTCTACTCGCAATCGAATCAACTACTATCGTACGGAGACGTAACAAGGTTCCTATCGAATAATACAATGGTACTATACACAGACGTATCGCAATGGGACTCGTCGCAACACAATACACAACCCTTCAGGAAGGGAATAATAATGGGACTAGACATACTAGCTAATATGACAAATGACGCTAAGGTACTACAAACACTAAATCTATACAAGCAAACACAAATAAATCTAATGGACTCGTACGTACAAATACCCGACGGAAATGTAATAAAGAAGATACAATACGGAGCTGTAGCTTCGGGAGAGAAGCAAACAAAGGCTGCTAATTCGATAGCTAATCTAGCTCTAATAAAGACAGTACTATCGAGGATATCGAATAAGCACTCGTTCGCTACAAAGATAATAAGGGTAGACGGAGACGACAATTACGCTGTACTACAATTCAATACAGAGGTAACAAAGCAAATGATACAAGACGTATCGAATGACGTAAGGGAGACATACGCTAGGATGAATGCTAAGGTAAAGGCTCTAGTATCGACAGTAGGAATAGAGATAGCTAAGAGGTACATAGCTGGAGGAAAGATATTCTTCAGGGCTGGAATAAATCTACTAAATAATGAGAAGAGGGGACAATCGACACAATGGGACCAAGCTGCTATACTATACTCGAATTACATAGTAAATAGGCTAAGGGGATTCGAGACAGACAGGGAGTTCATACTAACAAAGATAATGCAAATGACATCGGTAGCTATAACAGGATCGCTAAGGCTATTCCCCTCGGAGAGGGTACTAACAACAAATTCGACATTCAAGGTATTCGACTCGGAGGACTTCATAATAGAGTACGGAACAACAGACGACGAGGTATACATACAAAGGGCTTTCATGTCGCTATCGTCGCAAAAGTCGGGAATAGCTGACGAGATAGCTGCTTCGTCGACATTCAAGAATTACGTAACAAGGCTATCGGAGCAACTACTATTCTCGAAGAATAATATAGTATCGAGGGGAATAGCTCTAACAGAGAAGGCTAAGCTAAATTCGTACGCTCCCATATCGCTAGAGAAGAGGAGGGCTCAAATATCGGCTCTACTAACAATGCTACAAAAGCCCGTAACATTCAAGTCGTCGAAGATAACAATAAATGACATACTAAGGGACATAAAGCCCTTCTTCACAGTATCGGACGCTCACCTACCCATACAATACCAAAAGTTCATGCCCACACTACCCGACAATGTACAATACATAATACAATGTATAGGATCGAGGACATACCAAATAGAGGACGACGGATCGAAGTCGGCTATATCGAGGCTAATATCGAAGTACTCGGTATACAAGCCCTCGATAGAGGAGCTATACAAGGTAATATCGCTACACGAGAATGAGATACAACTATACCTAATATCGCTAGGAATACCCAAGATAGACGCTGACACATACGTAGGATCGAAGATATACTCGCAAGACAAGTACAGGATACTAGAGTCGTACGTATACAATCTACTATCGATAAATTACGGATGTTACCAACTATTCGACTTCAATTCGCCCGACCTAGAGAAGCTAATAAGGATACCCTTCAAGGGAAAGATACCCGCTGTTACATTCATATTACACTTATATGCAAAGCTAGAAGTTATAAACTACGCTATAAAAAATGGTTCATGGATAAGCCTATTTTGCAATTACCCTAAATCAGAAATGATAAAATTATGGAAGAAGATGTGGAACATCACGTCATTACGTTCGCCGTACACTAACGCGAACTTCTTTCAAGATTAG

>SA11 VP2cd

ATGGCGTATCGAAAACGTGGAGCGCGTCGTGAGACGAATCTAAAACAAGATGAACGAATGCAAGAAAAAGAAGATAGCAAGAACATTAATAATGACAGTCCTAAATCACAATTATCAGAAAAAGTATTATCTAAGAAAGAAGAGATAATTACAGATAATCAAGAAGAAGTTAAGATATCTGATGAGGTAAAGAAGTCGAATAAGGAGGAGTCGAAGCAACTACTAGAGGTACTAAAGACAAAGGAGGAGCACCAAAAGGAGGTACAATACGAGATACTACAAAAGACAATACCCACATTCGAGCCCAAGGAGTCGATACTAAAGAAGCTAGAGGACATAAAGCCCGAGCAAGCTAAGAAGCAAACAAAGCTATTCAGGATATTCGAGCCCAAGCAACTACCCATATACAGGGCTAATGGAGAGAGGGAGCTAAGGAATAGGTGGTACTGGAAGCTAAAGAGGGACACACTACCCGACGGaGACTACGACGTAAGGGAGTACTTCCTAAATCTATACGACCAAGTACTAATGGAGATGCCCGACTACCTACTACTAAAGGACATGGcTGTAgAGAATAAGAATTCGAGGGACGCTGGAAAGGtaGTAGACTCGGAGACaGCTGCTaTATGTGACGCTATATTCcaaGACGAGGAGACAGAGGGAGCTGTAAGGAGGTTCaTAgCTGAgAtGAGGCAaAGGGTAcaagcTGACAGGAATGtAGTAaATTACCCCTCGATACTACACCCCATAGacCACGCTTTCAATGAGtACTTCCTACAACACCAACTAGTAGAGCCCCTAAATAATGACATAATATTCAATTACaTACCCgAGAGGATAAGGAATGACGTAAATTACATACTAAATATGGACAGGAATCTACCCTCGACAGCTAGGTACATAAGGCCCAATCTACTACAAGACAGGCTAAATCTACACGACAATTTCGAGTCGCTATGGGACACAATaACAACATCGAATTACATACTAGCTAGGTCGGTAGTACCCgACCTAAAGGAGCTAGTATCGACAGAGGCTCAAATACAAAAGATGTCGCAAGACCTACAACTAGAGGCTCTAACaATACAATCGGAGACACAATTCCTAACAGGAataAATTCGCAAGCTGCTAATGACTGTTTCAAGACACTAATAGcTGCTATGCTATCGCAAAGGACAATGTCGCTAGACTTCGTAACAACaaATTACaTGTCGCTAATATCGGGAATGTGGCTACTAACAGTAATACCCaaTGACATGTTCATAAGGGAGTCGCTAGTAGCTTGTCAACTAGCTaTAATAAATACAATAGTATACCCCGCTttcggaatgCAaAGGATGCACTACAGGAATGGAGACCCCCAAACACCCTTCCAAATAGCTGAgCaaCAAATACaAAATTTCCAAGTAgCTaaTtGgCTACACTTCGTAAATTACAATCAATTCAGGCAAGTAGTAATAGACGGAGTACTAAAtCaaGTACTAAATGACAAtATaAGGAATGGACACGTAGTAAATCAaCTAatGGAGgCTCTAAtGCAACTATCGAGGCAACAATTCCCCACAATGCCCGTAGACTACAAGAGGTCGATACAAAGGGGAATACTACTACTATCGAATAGGCTAGGACAACTAGTAGACCTAACAAGGCTACTATCGTACAATTACGAGACACTAATGGCTTGTATAACAATGAATATGCAACACGTACAAACACTAACAACAGAGAAGCTACAACTAACATCGGTAACATCGCTATGTATGCTAATAGGAAATGCTACAGTAATACCCTCGCCCCAAACACTATTCCACTACTACAATGTAAATGTAAATTTCCACTCGAATTACAATGAGAGGATAAATGACGCTGTAGCTATAATAACAGCTGCTAATAGGCTAAATCTATACCAAAAGAAGATGAAGTCGATAGTAGAGGACTTCCTAAAGAGGCTACAAATATTCGACGTAGCTAGGGTACCCGACGACCAAATGTACAGGCTAAGGGACAGGCTAAGGCTACTACCCGTAGAGATAAGGAGGCTAGACATATTCAATCTAATAGCTATGAATATGGAGCAAATAGAGAGGGCTTCGGACAAGATAGCTCAAGGAGTAATAATAGCTTACAGGGACATGCAACTAGAGAGGGACGAGATGTACGGATACGTAAATATAGCTAGGAATCTAGACGGATTCCAACAAATAAATCTAGAGGAGCTAATGAGGTCGGGAGACTACGCTCAAATAACAAATATGCTACTAAATAATCAACCCGTAGCTCTAGTAGGAGCTCTACCCTTCATAACAGACTCGTCGGTAATATCGCTAATAGCTAAGCTAGACGCTACAGTATTCGCTCAAATAGTAAAGCTAAGGAAGGTAGACACACTAAAGCCCATACTATACAAGATAAATTCGGACTCGAATGACTTCTACCTAGTAGCTAATTACGACTGGATACCCACATCGACAACAAAAGTGTATAAACAAGTTCCACAACAATTTGATTTTAGAGCGTCAATGCATATGTTAACGTCTAACCTAACATTTACCGTATATTCAGATTTGCTTGCGTTCGTTTCAGCTGATACTGTTGAACCAATTAATGCTGTTGCTTTTGATAATATGCGCATCATGAACGAACTGTAA

>SA11 VP3cd

ATGAAAGTACTAGCTTTAAGACACAGTGTGGCTCAAGTGTATGCAGACACTCAAGTCTACGTTCATGATGATACAAAAGATAGTTATGAAAACGCTTTTTTAATCTCTAATCTTACGAcCCATaaTATTTTATACTTAAATTATAgCaTTaaAACACTAGAGATACTAAATAAGTCGGGAATAGCTGCTATAGCTCTACAATCGCTAGAGGAGCTATTCACACTAATAAGGTGTAATTTCACATACGACTACGAGCTAGACATAATATACCTACACGACTACTCGTACTACACAAATAATGAGATAAGGACAGACCAaCACTGGATAACAAAGACAAATATAGAGGAGTACCTACTACCCGGATGGAAGCTAACATACGTAGGATACAATGGATCGGAGACAAGGGGACACTACAATTTCTCGTTCAAGTGTCAAAATGCTGCTACAGACGACGACCTAATAATAGAGTACATATACTCGGAGGCTCTAGACTTCCAAAATTTCATGCTAAAGAAGATAAAGGAGAGGATGACAACATCGCTACCCATAGCTAGGCTATCGAATAGGGTATTCAGGGACAAGCTATTCCCCTCGCTACTAAAGGAGCACAAGAATGTAGTAAATGTAGGACCCAGGAATGAGTCGATGTTCACATTCCTAAATTACCCCACAATAAAGCAATTCTCGAATGGAGCTTACCTAGTAAAGGACACAATAAAGCTAAAGCAAGAGAGGTGGCTAGGAAAGAGGATATCGCAATTCGACATAGGACAATACAAGAATATGCTAAATGTACTAACAGCTATATACTACTACTACAATCTATACAAGTCGAAGCCCATAATATACATGATAGGATCGGCTCCCTCGTACTGGATATACGACGTAAGGCACTACTCGGACTTCTTCTTCGAGACATGGGACCCCCTAGACACACCCTACTCGTCGATACACCACAAGGAGCTATTCTTCATAAATGACGTAAAGAAGCTAAAGGACAATTCGATACTATACATAGACATAAGGACAGACAGGGGAAATGCTGACTGGAAGAAGTGGAGGAAGACAGTAGAGGAGCAAACAATAAATAATCTAGACATAGCTTACGAGTACCTAAGGACAGGAAAGGCTAAGGTATGTTGTGTAAAGATGACAGCTATGGACCTAGAGCTACCCATATCGGCTAAGCTACTACACCACCCCACAACAGAGATAAGGTCGGAGTTCTACCTACTACTAGACACATGGGACCTAACAAATATAAGGAGGTTCATACCCAAGGGAGTACTATACTCGTTCATAAATAATATAATAACAGAGAATGTATTCATACAACAACCCTTCAAGGTAAAGGTACTAAATGACTCGTACATAGTAGCTCTATACGCTCTATCGaaTGACTTCAATAaTAGGTCGGAGgtaATAAAGCTAATAAaTAATCAAAAGCaATCGCTAATaACAGTAAGGATAAATAATACATTCAAGGACGAGCCCAAGGTAGGATTCAAGAATATATACGACtGGACATTCCTACCCACAgACTTCGACACAAAGGAGgcTaTAATAACATCGTACGACGGATGTCTAGGACTATTCGGACTATCGATaTCGCTAGCTTCGAAGCCCACAGGAAATAATCACCTATTCATACTATCGGGAACAGACAAGTACTACAAGCTAGACCAATTCGCTAATCaCACATCGATATCGAGGAGGTCGCACCAAATAAGGTTCTCGGAGTCGGCTACATCGTACTCGGGATACATATTCAGGGACCTATCGAATAATAATTTCAATCTAATAGGAACAAAtATAGAGAATTCGGTATCGGGACACGtATACaATGcTCTAATATACTACAGGTACAATTACTCGTTCGACCTAAAGAGGTGGATATACCTACACTCGATAGACAAGGTAGACATAGAGGGAgGAAAGTACTACGAGCACGCTCCCATAGAGCTAATATACGCTTGTAGGTCGGCTAAGGAGTTCGCTACACTACAAGACGACCTAACAGTACTAAGGTACTCGAATGAGATAGAGAATTACATAAATACAGTATACTCGATAACATACGCTGACGACCCCAATTACTTCATAGGAATACAATTCAGGAATATACCCTACAAGTACGACGTAAAGATACCCCACCTAACATTCGGAGTACTACACATATCGGACAATATGGTACCCGACGTAATAGACATACTAAAGATAATGAAGAATGAGCTATTCAAGATGGACATAACAACATCGTACACATACATGCTATCGGACGGAATATACGTAGCTAATGTATCGGGAGTACTATCGACATACTTCAAGATATACAATGTATTTTATAAAAATCAAATAACTTTTGGCCAATCCAGAATGTTTATTCCGCACATAACATTAAGCTTCAATAACATGAGAACAGTAAGGATAGAGACTACTAAATTACAAATTAAATCCATTTATTTAAGAAAGATTAAGGGTGATACAGTGTTTGATATGGTTGAGTGA

>SA11 VP6cd

ATGGATGTCCTATACTCTTTGTCAAAGACTCTTAAAGACGCTAGAGACAAAATTGTCGAAGGCACATTGTATTCTAACGTGAGTGATCTAATTCAACAATTTAATCAAATGATAATTACTATGAATGGAAATGAATTTCAAACTGGAGGAATCGGTAATTTGCCAATTAGAAACTGGAATTTCAATTTCGGACTACTAGGAACAACACTACTAAATCTAGACGCTAATTACGTAGAGACAGCTAGGAATACAATAGACTACTTCGTAGACTTCGTAGACAATGTATGTATGGACGAGATGGTAAGGGAGTCGCAAAGGAATGGAATAGCTCCCCAATCGGACTCGCTAAGGAAGCTATCGGCTATAAAGTTCAAGAGGATAAATTTCGACAATTCGTCGGAGTACATAGAGAATTGGAATCTACAAAATAGGAGGCAAAGGACAGGATTCACATTCCACAAGCCCAATATATTCCCCTACTCGGCTTCGTTCACACTAAATAGGTCGCAACCCGCTCACGACAATCTAATGGGAACAATGTGGCTAAATGCTGGATCGGAGATACAAGTAGCTGGATTCGACTACTCGTGTGCTATAAATGCTCCCGCTAATATACAACAATTCGAGCACATAGTACCCCTAAGGAGGGTACTAACAACAGCTACAATAACACTACTACCCGACGCTGAGAGGTTCTCGTTCCCCAGGGTAATAAATTCGGCTGACGGAGCTACAACATGGTTCTTCAATCCCGTAATACTAAGGCCCAATAATGTAGAGGTAGAGTTCCTACTAAATGGACAAATAATAAATACATACCAAGCTAGGTTCGGAACAATAGTAGCTAGGAATTTCGACACAATAAGGCTATCGTTCCAACTAATGAGGCCCCCCAATATGACACCCGCTGTAGCTGTACTATTCCCCAATGCTCAACCCTTCGAGCACCACGCTACAGTAGGACTAACACTAAGGATAGAGTCGGCTGTATGTGAGTCGGTACTAGCTGACGCTTCGGAGACACTACTAGCTAATGTAACATCGGTAAGGCAAGAGTACGCTATACCCGTAGGACCCGTATTCCCCCCCGGAATGAATTGGACAGACCTAATAACAAATTACTCGCCCTCGAGGGAGGACAATTTGCAACGCGTATTTACAGTGGCTTCCATTAGAAGCATGCTCATTAAATGA

>SA11 NSP2cd

ATGGCTGAGCTAGCTTGCTTTTGCTATCCTCATTTGGAGAATGATAGCTATAAATTCATACCCTTCAATAATCTAGCTATAAAGGCTATGCTAACAGCTAAGGTAGACAAGAAGGACATGGACAAGTTCTACGACTCGATAATATACGGAATAGCTCCCCCCCCCCAATTCAAGAAGAGGTACAATACAAATGACAATTCGAGGGGAATGAATTTCGAGACAATAATGTTCACAAAGGTAGCTATGCTAATATGTGAGGCTCTAAATTCGCTAAAGGTAACACAAGCTAATGTATCGAATGTACTATCGAGGGTAGTATCGATAAGGCACCTAGAGAATCTAGTAATAAGGAAGGAGAATCCCCAAGACATACTATTCCACTCGAAGGACCTACTACTAAAGTCGACACTAATAGCTATAGGACAATCGAAGGAGATAGAGACAACAATAACAGCTGAGGGAGGAGAGATAGTATTCCAAAATGCTGCTTTCACAATGTGGAAGCTAACATACCTAGAGCACCAACTAATGCCCATACTAGACCAAAATTTCATAGAGTACAAGGTAACACTAAATGAGGACAAGCCCATATCGGACGTACACGTAAAGGAGCTAGTAGCTGAGCTAAGGTGGCAATACAATAAGTTCGCTGTAATAACACACGGAAAGGGACACTACAGGATAGTAAAGTACTCGTCGGTAGCTAATCACGCTGACAGGGTATACGCTACATTCAAGTCGAATGTAAAGACAGGAGTAAATAATGACTTCAATCTACTAGACCAAAGGATAATATGGCAAAATTGGTACGCTTTCACATCGTCGATGAAGCAAGGAAATACACTAGACGTATGTAAGAGGCTACTATTCCAAAAGATGAAGCCCGAGAAGAATCCCTTCAAGGGACTATCGACGGATAGAAAAATGGACGAAGTTTCTCAAGTTGGCGTTTAA

>SA11 NSP3cd

AtgctcaagatggagtctacgcaacagatggccgtctcaattattaactcttcttttgaagctgcagttgtagctGCTACATCGGCTCTAGAGaatatgggaataGAGTACGACTACCAAGACataTACTCGAGGGTAAAGaatAAGTTCGACTTCGTAatgGACGACTCGGGAGTAAAGaataatCTAATAGGAAAGGCTataACAATAGACcaaGCTCTAaataatAAGTTCGGATCGgctataAGGaatAGGAATtggCTAgctGACACATCGAGGGCTgctAAGCTAGACGAGGACgtaAATAAGCTAAGGatgatgCTATCGTCGAAGggaATAGACcaaAAGatgAGGGTACTAAATGCTTGTttcTCGgtaAAGAGGataCCCggaAAGTCGTCGTCGATAATAAAGTGTACAAAGCTAatgAGGGACAAGCTAGAGAGGGGAGAGGTAGAGGTAGACGACTCGTTCGTAGACGAGAAGatgGAGGTAGACACAATAgactggAAGTCGAGGTACgagcaaCTAgagcaaaggTTCGAGTCGCTAAAGTCGagggtaaatGAGAAGTACaataattggGTACTAAAGGCTAGGAAGatgaatGAGaatatgCACTCGCTAcaaaatGTAATATCGcaaCAAcaaGCTCACatagctgagCTAcaaGTAtacaataatAAGctaGAGAGGGACCTAcaaaatAAGATAggaTCGCTAACATCGTCGATAGAGtggTACCTAAGGTCGatgGAGCTAgacCCCGAGATAAAGGCTGACATAGAGCAAcaaATAAATTCGATAGACGCTataaatCCCCTAcacgctTTCGACgacCTAGAGTCGgtaataAGGaatCTAataTCGGACTACgacAAGCTAttcCTAatgttcAAGggaCTAataCAAAGGTGTaatTACcaaTACTCGTTCGGATGTGAGtaa

>SA11 NSP4cd

ATGGAAAAGCTTACCGACCTCAATTATACATTGAGTGTAATCACTCTAATGAACAATACACTACACACAATACTAGAGGACCCCGGAATGGCTTACTTCCCCTACATAGCTTCGGTACTAACAGTACTATTCGCTCTACACAAGGCTTCGATACCCACAATGAAGATAGCTCTAAAGACATCGAAGTGTTCGTACAAGGTAGTAAAGTACTGTATAGTAACAATATTCAATACACTACTAAAGCTAGCTGGATACAAGGAGCAAATAACAACAAAGGACGAGATAGAGAAGCAAATGGACAGGGTAGTAAAGGAGATGAGGAGGCAACTAGAGATGATAGACAAGCTAACAACAAGGGAGATAGAGCAAGTAGAGCTACTAAAGAGGATATACGACAAGCTAACAGTACAAACAACAGGAGAGATAGACATGACAAAGGAGATAAATCAAAAGAATGTAAGGACACTAGAGGAGTGGGAGTCGGGAAAGAATCCCTACGAGCCCAGGGAGGTAACAGCTGCTATGTAA

>SA11 NSP5cd

ATGTCTCTCAGTATTGACGTGACGAGTCTTCCTTCTATTCCTTCAACTATATATAAGAATGAATCGTCTTCAACAACGTCAACACTATCGGGAAAGTCGATAGGAAGGTCGGAGCAATACATATCGCCCGACGCTGAGGCTTTCAATAAGTACATGCTATCGAAGTCGCCCGAGGACATAGGACCCTCGGACTCGGCTTCGAATGACCCCCTAACATCGTTCTCGATAAGGTCGAATGCTGTAAAGACAAATGCTGACGCTGGAGTATCGATGGACTCGTCGGCTCAATCGAGGCCCTCGTCGAATGTAGGATGTGACCAAGTAGACTTCTCGCTAAATAAGGGACTAAAGGTAAAGGCTAATCTAGACTCGTCGATATCGATATCGACAGACACAAAGAAGGAGAAGTCGAAGCAAAATCACAAGTCGAGGAAGCACTACCCCAGGATAGAGGCTGAGTCGGACTCGGACGACTACGTACTAGACGACTCGGACTCGGACGACGGAAAGTGTAAGAATTGTAAGTACAAGAAGAAGTACTTCGCTTTAAGAATGAGAATGAAACAAGTCGCAATGCAATTGATTGAAGATTTGTAA

>SA11 VP1 high GC

GGCTATTAAAGCTGTACAATGGGGAAGTACAATCTAATCTTGTCAGAATATCTATCATTTATATATAATTCACAATCTGCAGTTCAAATTCCAATATATTACTCTTCCAACAGTGAATTAGAAAATAGATGTATTGAATTTCATTCCAAGTGTTTAGAGAACTCAAAGAATGGGTTATCGTTAAGAAAGTTGTTTGTTGAATATAACGATGTGATCGAAAACGCGACGCTGCTGAGCATCCTGAGCTATAGCTATGATAAATATAACGCGGTGGAACGCAAACTGGTGAAATATGCGAAAGGCAAACCGCTGGAAGCGGATCTGACGGTGAACGAACTGGATTATGAAAACAACAAAATCACGAGCGAACTGTTTCCGACGGCGGAAGAATATACGGATAGCCTGATGGATCCGGCGATCCTGACGAGCCTGAGCAGCAACCTGAACGCGGTGATGTTTTGGCTGGAAAAACATGAAAACGATGTGGCGGAAAAACTGAAAGTGTATAAACGCCGCCTGGATCTGTTTACGATCGTGGCGAGCACGATCAACAAATATGGCGTGCCGCGCCATAACGCGAAATATCGCTATGAATATGATGTGATGAAAGATAAACCGTATTATCTGGTGACGTGGGCGAACAGCAGCATCGAAATGCTGATGAGCGTGTTTAGCCATGATGATTATCTGATCGCGAAAGAACTGATCGTGCTGAGCTATAGCAACCGCAGCACGCTGGCGAAACTGGTGAGCAGCCCGATGAGCATCCTGGTGGCGCTGGTGGATATCAACGGCACGTTTATCACGAACGAAGAACTGGAACTGGAATTTAGCAACAAATATGTGCGCGCGATCGTGCCGGATCAGACGTTTGATGAACTGAACCAGATGCTGGATAACATGCGCAAAGCGGGCCTGGTGGATATCCCGAAAATGATCCAGGATTGGCTGGTGGATCGCAGCATCGAAAAATTTCCGCTGATGGCGAAAATCTATAGCTGGAGCTTTCATGTGGGCTTTCGCAAACAGAAAATGCTGGATGCGGCGCTGGATCAGCTGAAAACGGAATATACGGAAAACGTGGATGATGAAATGTATCGCGAATATACGATGCTGATCCGCGATGAAGTGGTGAAAATGCTGGAAGAACCGGTGAAACATGATGATCATCTGCTGCGCGATAGCGAACTGGCGGGCCTGCTGAGCATGAGCAGCGCGAGCAACGGCGAAAGCCGCCAGCTGAAATTTGGCCGCAAAACGATCTTTAGCACGAAAAAAAACATGCATGTGATGGATGATATGGCGAACGAACGCTATACGCCGGGCATCATCCCGCCGGTGAACGTGGATAAACCGATCCCGCTGGGCCGCCGCGATGTGCCGGGCCGCCGCACGCGCATCATCTTTATCCTGCCGTATGAATATTTTATCGCGCAGCATGCGGTGGTGGAAAAAATGCTGATCTATGCGAAACATACGCGCGAATATGCGGAATTTTATAGCCAGAGCAACCAGCTGCTGAGCTATGGCGATGTGACGCGCTTTCTGAGCAACAACACGATGGTGCTGTATACGGATGTGAGCCAGTGGGATAGCAGCCAGCATAACACGCAGCCGTTTCGCAAAGGCATCATCATGGGCCTGGATATCCTGGCGAACATGACGAACGATGCGAAAGTGCTGCAGACGCTGAACCTGTATAAACAGACGCAGATCAACCTGATGGATAGCTATGTGCAGATCCCGGATGGCAACGTGATCAAAAAAATCCAGTATGGCGCGGTGGCGAGCGGCGAAAAACAGACGAAAGCGGCGAACAGCATCGCGAACCTGGCGCTGATCAAAACGGTGCTGAGCCGCATCAGCAACAAACATAGCTTTGCGACGAAAATCATCCGCGTGGATGGCGATGATAACTATGCGGTGCTGCAGTTTAACACGGAAGTGACGAAACAGATGATCCAGGATGTGAGCAACGATGTGCGCGAAACGTATGCGCGCATGAACGCGAAAGTGAAAGCGCTGGTGAGCACGGTGGGCATCGAAATCGCGAAACGCTATATCGCGGGCGGCAAAATCTTTTTTCGCGCGGGCATCAACCTGCTGAACAACGAAAAACGCGGCCAGAGCACGCAGTGGGATCAGGCGGCGATCCTGTATAGCAACTATATCGTGAACCGCCTGCGCGGCTTTGAAACGGATCGCGAATTTATCCTGACGAAAATCATGCAGATGACGAGCGTGGCGATCACGGGCAGCCTGCGCCTGTTTCCGAGCGAACGCGTGCTGACGACGAACAGCACGTTTAAAGTGTTTGATAGCGAAGATTTTATCATCGAATATGGCACGACGGATGATGAAGTGTATATCCAGCGCGCGTTTATGAGCCTGAGCAGCCAGAAAAGCGGCATCGCGGATGAAATCGCGGCGAGCAGCACGTTTAAAAACTATGTGACGCGCCTGAGCGAACAGCTGCTGTTTAGCAAAAACAACATCGTGAGCCGCGGCATCGCGCTGACGGAAAAAGCGAAACTGAACAGCTATGCGCCGATCAGCCTGGAAAAACGCCGCGCGCAGATCAGCGCGCTGCTGACGATGCTGCAGAAACCGGTGACGTTTAAAAGCAGCAAAATCACGATCAACGATATCCTGCGCGATATCAAACCGTTTTTTACGGTGAGCGATGCGCATCTGCCGATCCAGTATCAGAAATTTATGCCGACGCTGCCGGATAACGTGCAGTATATCATCCAGTGCATCGGCAGCCGCACGTATCAGATCGAAGATGATGGCAGCAAAAGCGCGATCAGCCGCCTGATCAGCAAATATAGCGTGTATAAACCGAGCATCGAAGAACTGTATAAAGTGATCAGCCTGCATGAAAACGAAATCCAGCTGTATCTGATCAGCCTGGGCATCCCGAAAATCGATGCGGATACGTATGTGGGCAGCAAAATCTATAGCCAGGATAAATATCGCATCCTGGAAAGCTATGTGTATAACCTGCTGAGCATCAACTATGGCTGCTATCAGCTGTTTGATTTTAACAGCCCGGATCTGGAAAAACTGATCCGCATCCCGTTTAAAGGCAAAATCCCGGCGGTTACATTCATATTACACTTATATGCAAAGCTAGAAGTTATAAACTACGCTATAAAAAATGGTTCATGGATAAGCCTATTTTGCAATTACCCTAAATCAGAAATGATAAAATTATGGAAGAAGATGTGGAACATCACGTCATTACGTTCGCCGTACACTAACGCGAACTTCTTTCAAGATTAGAACGCTTAGATGTGACC

>SA11 VP2 high GC

GGCTATTAAAGGCTCAATGGCGTATCGAAAACGTGGAGCGCGTCGTGAGACGAATCTAAAACAAGATGAACGAATGCAAGAAAAAGAAGATAGCAAGAACATTAATAATGACAGTCCTAAATCACAATTATCAGAAAAAGTATTATCTAAGAAAGAAGAGATAATTACAGATAATCAAGAAGAAGTTAAGATATCTGATGAGGTGAAAAAAAGCAACAAAGAAGAAAGCAAACAGCTGCTGGAAGTGCTGAAAACGAAAGAAGAACATCAGAAAGAAGTGCAGTATGAAATCCTGCAGAAAACGATCCCGACGTTTGAACCGAAAGAAAGCATCCTGAAAAAACTGGAAGATATCAAACCGGAACAGGCGAAAAAACAGACGAAACTGTTTCGCATCTTTGAACCGAAACAGCTGCCGATCTATCGCGCGAACGGCGAACGCGAACTGCGCAACCGCTGGTATTGGAAACTGAAACGCGATACGCTGCCGGATGGCGATTATGATGTGCGCGAATATTTTCTGAACCTGTATGATCAGGTGCTGATGGAAATGCCGGATTATCTGCTGCTGAAAGATATGGCGGTGGAAAACAAAAACAGCCGCGATGCGGGCAAAGTGGTGGATAGCGAAACGGCGGCGATCTGCGATGCGATCTTTCAGGATGAAGAAACGGAAGGCGCGGTGCGCCGCTTTATCGCGGAAAtGCGCCAGCGCGTGCAGGCGGATCGCAACGTGGTGAACTATCCGAGCATCCTGCATCCGATCGATCATGCGTTTAACGAATATTTTCTGCAGCATCAGCTGGTGGAACCGCTGAACAACGATATCATCTTTAACTATATCCCGGAACGCATCCGCAACGATGTGAACTATATCCTGAACATGGATCGCAACCTGCCGAGCACGGCGCGCTATATCCGCCCGAACCTGCTGCAGGATCGCCTGAACCTGCATGATAACTTTGAAAGCCTGTGGGATACGATCACGACGAGCAACTATATCCTGGCGCGCAGCGTGGTGCCGGATCTGAAAGAACTGGTGAGCACGGAAGCGCAGATCCAGAAAATGAGCCAGGATCTGCAGCTGGAAGCGCTGACGATCCAGAGCGAAACGCAGTTTCTGACGGGCATCAACAGCCAGGCGGCGAACGATTGCTTTAAAACGCTGATCGCGGCGATGCTGAGCCAGCGCACGATGAGCCTGGATTTTGTGACGACGAACTATaTGAGCCTGATCAGCGGCATGTGGCTGCTGACGGTGATCCCGAACGATATGTTTATCCGCGAAAGCCTGGTGGCGTGCCAGCTGGCGATCATCAACACGATCGTGTATCCGGCGTTTGGCatgCAGCGCATGCATTATCGCAACGGCGATCCGCAGACGCCGTTTCAGATCGCGGAACAGCAGATCCAGAACTTTCAGGTGGCGAACtGgCTGCATTTTGTGAACTATAACCAGTTTCGCCAGGTGGTGATCGATGGCGTGCTGAACCAGGTGCTGAACGATAACATCCGCAACGGCCATGTGGTGAACCAGCTGatGGAAGCGCTGAtGCAGCTGAGCCGCCAGCAGTTTCCGACGATGCCGGTGGATTATAAACGCAGCATCCAGCGCGGCATCCTGCTGCTGAGCAACCGCCTGGGCCAGCTGGTGGATCTGACGCGCCTGCTGAGCTATAACTATGAAACGCTGATGGCGTGCATCACGATGAACATGCAGCATGTGCAGACGCTGACGACGGAAAAACTGCAGCTGACGAGCGTGACGAGCCTGTGCATGCTGATCGGCAACGCGACGGTGATCCCGAGCCCGCAGACGCTGTTTCATTATTATAACGTGAACGTGAACTTTCATAGCAACTATAACGAACGCATCAACGATGCGGTGGCGATCATCACGGCGGCGAACCGCCTGAACCTGTATCAGAAAAAAATGAAAAGCATCGTGGAAGATTTTCTGAAACGCCTGCAGATCTTTGATGTGGCGCGCGTGCCGGATGATCAGATGTATCGCCTGCGCGATCGCCTGCGCCTGCTGCCGGTGGAAATCCGCCGCCTGGATATCTTTAACCTGATCGCGATGAACATGGAACAGATCGAACGCGCGAGCGATAAAATCGCGCAGGGCGTGATCATCGCGTATCGCGATATGCAGCTGGAACGCGATGAAATGTATGGCTATGTGAACATCGCGCGCAACCTGGATGGCTTTCAGCAGATCAACCTGGAAGAACTGATGCGCAGCGGCGATTATGCGCAGATCACGAACATGCTGCTGAACAACCAGCCGGTGGCGCTGGTGGGCGCGCTGCCGTTTATCACGGATAGCAGCGTGATCAGCCTGATCGCGAAACTGGATGCGACGGTGTTTGCGCAGATCGTGAAACTGCGCAAAGTGGATACGCTGAAACCGATCCTGTATAAAATCAACAGCGATAGCAACGATTTTTATCTGGTGGCGAACTATGATTGGATCCCGACGAGCACGACGAAAGTGTATAAACAAGTTCCACAACAATTTGATTTTAGAGCGTCAATGCATATGTTAACGTCTAACCTAACATTTACCGTATATTCAGATTTGCTTGCGTTCGTTTCAGCTGATACTGTTGAACCAATTAATGCTGTTGCTTTTGATAATATGCGCATCATGAACGAACTGTAAACGCCAACCCCATTGTGGAGATATGACC

>SA11 VP3 high GC

GGCTATTAAAGCAGTACCAGTAGTGTGTTTTACCTCTGATGGTGTAAACATGAAAGTACTAGCTTTAAGACACAGTGTGGCTCAAGTGTATGCAGACACTCAAGTCTACGTTCATGATGATACAAAAGATAGTTATGAAAACGCTTTTTTAATCTCTAATCTTACGAcCCATaaTATTTTATACTTAAATTATAgCaTTaaAACGCTGGAAATCCTGAACAAAAGCGGCATCGCGGCGATCGCGCTGCAGAGCCTGGAAGAACTGTTTACGCTGATCCGCTGCAACTTTACGTATGATTATGAACTGGATATCATCTATCTGCATGATTATAGCTATTATACGAACAACGAAATCCGCACGGATCAGCATTGGATCACGAAAACGAACATCGAAGAATATCTGCTGCCGGGCTGGAAACTGACGTATGTGGGCTATAACGGCAGCGAAACGCGCGGCCATTATAACTTTAGCTTTAAATGCCAGAACGCGGCGACGGATGATGATCTGATCATCGAATATATCTATAGCGAAGCGCTGGATTTTCAGAACTTTATGCTGAAAAAAATCAAAGAACGCATGACGACGAGCCTGCCGATCGCGCGCCTGAGCAACCGCGTGTTTCGCGATAAACTGTTTCCGAGCCTGCTGAAAGAACATAAAAACGTGGTGAACGTGGGCCCGCGCAACGAAAGCATGTTTACGTTTCTGAACTATCCGACGATCAAACAGTTTAGCAACGGCGCGTATCTGGTGAAAGATACGATCAAACTGAAACAGGAACGCTGGCTGGGCAAACGCATCAGCCAGTTTGATATCGGCCAGTATAAAAACATGCTGAACGTGCTGACGGCGATCTATTATTATTATAACCTGTATAAAAGCAAACCGATCATCTATATGATCGGCAGCGCGCCGAGCTATTGGATCTATGATGTGCGCCATTATAGCGATTTTTTTTTTGAAACGTGGGATCCGCTGGATACGCCGTATAGCAGCATCCATCATAAAGAACTGTTTTTTATCAACGATGTGAAAAAACTGAAAGATAACAGCATCCTGTATATCGATATCCGCACGGATCGCGGCAACGCGGATTGGAAAAAATGGCGCAAAACGGTGGAAGAACAGACGATCAACAACCTGGATATCGCGTATGAATATCTGCGCACGGGCAAAGCGAAAGTGTGCTGCGTGAAAATGACGGCGATGGATCTGGAACTGCCGATCAGCGCGAAACTGCTGCATCATCCGACGACGGAAATCCGCAGCGAATTTTATCTGCTGCTGGATACGTGGGATCTGACGAACATCCGCCGCTTTATCCCGAAAGGCGTGCTGTATAGCTTTATCAACAACATCATCACGGAAAACGTGTTTATCCAGCAGCCGTTTAAAGTGAAAGTGCTGAACGATAGCTATATCGTGGCGCTGTATGCGCTGAGCAACGATTTTAACAACCGCAGCGAAGTGATCAAACTGATCAACAACCAGAAACAGAGCCTGATCACGGTGCGCATCAACAACACGTTTAAAGATGAACCGAAAGTGGGCTTTAAAAACATCTATGATtGGACGTTTCTGCCGACGGATTTTGATACGAAAGAAGCGATCATCACGAGCTATGATGGCTGCCTGGGCCTGTTTGGCCTGAGCATCAGCCTGGCGAGCAAACCGACGGGCAACAACCATCTGTTTATCCTGAGCGGCACGGATAAATATTATAAACTGGATCAGTTTGCGAACCATACGAGCATCAGCCGCCGCAGCCATCAGATCCGCTTTAGCGAAAGCGCGACGAGCTATAGCGGCTATATCTTTCGCGATCTGAGCAACAACAACTTTAACCTGATCGGCACGAACATCGAAAACAGCGTGAGCGGCCATGTGTATAACGCGCTGATCTATTATCGCTATAACTATAGCTTTGATCTGAAACGCTGGATCTATCTGCATAGCATCGATAAAGTGGATATCGAAGGCGGCAAATATTATGAACATGCGCCGATCGAACTGATCTATGCGTGCCGCAGCGCGAAAGAATTTGCGACGCTGCAGGATGATCTGACGGTGCTGCGCTATAGCAACGAAATCGAAAACTATATCAACACGGTGTATAGCATCACGTATGCGGATGATCCGAACTATTTTATCGGCATCCAGTTTCGCAACATCCCGTATAAATATGATGTGAAAATCCCGCATCTGACGTTTGGCGTGCTGCATATCAGCGATAACATGGTGCCGGATGTGATCGATATCCTGAAAATCATGAAAAACGAACTGTTTAAAATGGATATCACGACGAGCTATACGTATATGCTGAGCGATGGCATCTATGTGGCGAACGTGAGCGGCGTGCTGAGCACGTATTTTAAAATCTATAACGTGTTTTATAAAAATCAAATAACTTTTGGCCAATCCAGAATGTTTATTCCGCACATAACATTAAGCTTCAATAACATGAGAACAGTAAGGATAGAGACTACTAAATTACAAATTAAATCCATTTATTTAAGAAAGATTAAGGGTGATACAGTGTTTGATATGGTTGAGTGAGCTAAAAACTTAACACACTAGTCATGATGTGACC

>SA11 VP6 high GC

GCTTTTAAACGAAGTCTTCAACATGGATGTCCTATACTCTTTGTCAAAGACTCTTAAAGACGCTAGAGACAAAATTGTCGAAGGCACATTGTATTCTAACGTGAGTGATCTAATTCAACAATTTAATCAAATGATAATTACTATGAATGGAAATGAATTTCAAACTGGAGGAATCGGTAATTTGCCAATTAGAAACTGGAACTTTAACTTTGGCCTGCTGGGCACGACGCTGCTGAACCTGGATGCGAACTATGTGGAAACGGCGCGCAACACGATCGATTATTTTGTGGATTTTGTGGATAACGTGTGCATGGATGAAATGGTGCGCGAAAGCCAGCGCAACGGCATCGCGCCGCAGAGCGATAGCCTGCGCAAACTGAGCGCGATCAAATTTAAACGCATCAACTTTGATAACAGCAGCGAATATATCGAAAACTGGAACCTGCAGAACCGCCGCCAGCGCACGGGCTTTACGTTTCATAAACCGAACATCTTTCCGTATAGCGCGAGCTTTACGCTGAACCGCAGCCAGCCGGCGCATGATAACCTGATGGGCACGATGTGGCTGAACGCGGGCAGCGAAATCCAGGTGGCGGGCTTTGATTATAGCTGCGCGATCAACGCGCCGGCGAACATCCAGCAGTTTGAACATATCGTGCCGCTGCGCCGCGTGCTGACGACGGCGACGATCACGCTGCTGCCGGATGCGGAACGCTTTAGCTTTCCGCGCGTGATCAACAGCGCGGATGGCGCGACGACGTGGTTTTTTAACCCGGTGATCCTGCGCCCGAACAACGTGGAAGTGGAATTTCTGCTGAACGGCCAGATCATCAACACGTATCAGGCGCGCTTTGGCACGATCGTGGCGCGCAACTTTGATACGATCCGCCTGAGCTTTCAGCTGATGCGCCCGCCGAACATGACGCCGGCGGTGGCGGTGCTGTTTCCGAACGCGCAGCCGTTTGAACATCATGCGACGGTGGGCCTGACGCTGCGCATCGAAAGCGCGGTGTGCGAAAGCGTGCTGGCGGATGCGAGCGAAACGCTGCTGGCGAACGTGACGAGCGTGCGCCAGGAATATGCGATCCCGGTGGGCCCGGTGTTTCCGCCGGGCATGAACTGGACGGATCTGATCACGAACTATAGCCCGAGCAGGGAGGACAATTTGCAACGCGTATTTACAGTGGCTTCCATTAGAAGCATGCTCATTAAATGAGGACCAAGCTAACAACTTGGTATCCAACTTTGGTGAGTATGTAGCTATATCAAGCTGTTTGAACTCTGTAAGTAAGGATGCGTATACGCATTCGCTACACAGAGTAATCACTCAGATGGTATAGTGAGAGGATGTGACC

>SA11 NSP2 high GC

GCTTTTAAAGCGTCTCAGTCGCCGTTTGAGCCTTGCGGTGTAGCCATGGCTGAGCTAGCTTGCTTTTGCTATCCTCATTTGGAGAATGATAGCTATAAATTTATCCCGTTTAACAACCTGGCGATCAAAGCGATGCTGACGGCGAAAGTGGATAAAAAAGATATGGATAAATTTTATGATAGCATCATCTATGGCATCGCGCCGCCGCCGCAGTTTAAAAAACGCTATAACACGAACGATAACAGCCGCGGCATGAACTTTGAAACGATCATGTTTACGAAAGTGGCGATGCTGATCTGCGAAGCGCTGAACAGCCTGAAAGTGACGCAGGCGAACGTGAGCAACGTGCTGAGCCGCGTGGTGAGCATCCGCCATCTGGAAAACCTGGTGATCCGCAAAGAAAACCCGCAGGATATCCTGTTTCATAGCAAAGATCTGCTGCTGAAAAGCACGCTGATCGCGATCGGCCAGAGCAAAGAAATCGAAACGACGATCACGGCGGAAGGCGGCGAAATCGTGTTTCAGAACGCGGCGTTTACGATGTGGAAACTGACGTATCTGGAACATCAGCTGATGCCGATCCTGGATCAGAACTTTATCGAATATAAAGTGACGCTGAACGAAGATAAACCGATCAGCGATGTGCATGTGAAAGAACTGGTGGCGGAACTGCGCTGGCAGTATAACAAATTTGCGGTGATCACGCATGGCAAAGGCCATTATCGCATCGTGAAATATAGCAGCGTGGCGAACCATGCGGATCGCGTGTATGCGACGTTTAAAAGCAACGTGAAAACGGGCGTGAACAACGATTTTAACCTGCTGGATCAGCGCATCATCTGGCAGAACTGGTATGCGTTTACGAGCAGCATGAAACAGGGCAACACGCTGGATGTGTGCAAACGCCTGCTGTTTCAGAAAATGAAACCGGAAAAAAACCCGTTTAAAGGCCTGAGCACGGATAGAAAAATGGACGAAGTTTCTCAAGTTGGCGTTTAATTCGCTATCAATTTGAGGATGATGATGGCTTAGCAAGAATAGAAAGCGCTTATGTGACC

>SA11 NSP3 high GC

ggcatttaatgcttttcagtggttgatgctcaagatggagtctacgcaacagatggccgtctcaattattaactcttcttttgaagctgcagttgtagctGCGACGAGCGCGCTGGAAAACatgGGCATCGAATATGATTATCAGGATATCTATAGCCGCGTGAAAAACAAATTTGATTTTGTGatgGATGATAGCGGCGTGAAAAACAACCTGATCGGCAAAGCGATCACGATCGATCAGGCGCTGAACAACAAATTTGGCAGCGCGATCCGCAACCGCAACtggCTGGCGGATACGAGCCGCGCGGCGAAACTGGATGAAGATGTGAACAAACTGCGCatgatgCTGAGCAGCAAAGGCATCGATCAGAAAatgCGCGTGCTGAACGCGTGCTTTAGCGTGAAACGCATCCCGGGCAAAAGCAGCAGCATCATCAAATGCACGAAACTGatgCGCGATAAACTGGAACGCGGCGAAGTGGAAGTGGATGATAGCTTTGTGGATGAAAAAatgGAAGTGGATACGATCGATtggAAAAGCCGCTATGAACAGCTGGAACAGCGCTTTGAAAGCCTGAAAAGCCGCGTGAACGAAAAATATAACAACtggGTGCTGAAAGCGCGCAAAatgAACGAAAACatgCATAGCCTGCAGAACGTGATCAGCCAGCAGCAGGCGCATATCGCGGAACTGCAGGTGTATAACAACAAACTGGAACGCGATCTGCAGAACAAAATCGGCAGCCTGACGAGCAGCATCGAAtggTATCTGCGCAGCatgGAACTGGATCCGGAAATCAAAGCGGATATCGAACAGCAGATCAACAGCATCGATGCGATCAACCCGCTGCATGCGTTTGATGATCTGGAAAGCGTGATCCGCAACCTGATCAGCGATTATGATAAACTGTTTCTGatgTTTAAAGGCCTGATCCAGCGCTGCAACTATCAGTATAGCTTTGGCTGCGAAtaaccattttgatacatgttgaacaatcaaatacagtgttagtatgttgtcatctatgcataaccctctatgagcacaatagttaaaagctaacactgtcaaaaacctaaatggctataggggcgttatgtggcc

>SA11 NSP4 high GC

GCTTTTAAAAGTTCTGTTCCGAGAGAGCGCGTGCGGAAAGATGGAAAAGCTTACCGACCTCAATTATACATTGAGTGTAATCACTCTAATGAACAATACACTGCATACGATCCTGGAAGATCCGGGCATGGCGTATTTTCCGTATATCGCGAGCGTGCTGACGGTGCTGTTTGCGCTGCATAAAGCGAGCATCCCGACGATGAAAATCGCGCTGAAAACGAGCAAATGCAGCTATAAAGTGGTGAAATATTGCATCGTGACGATCTTTAACACGCTGCTGAAACTGGCGGGCTATAAAGAACAGATCACGACGAAAGATGAAATCGAAAAACAGATGGATCGCGTGGTGAAAGAAATGCGCCGCCAGCTGGAAATGATCGATAAACTGACGACGCGCGAAATCGAACAGGTGGAACTGCTGAAACGCATCTATGATAAACTGACGGTGCAGACGACGGGCGAAATCGATATGACGAAAGAAATCAACCAGAAAAACGTGCGCACGCTGGAAGAATGGGAAAGCGGCAAAAACCCGTATGAACCGCGCGAAGTGACGGCGGCGATGTAAGAGGTTGAGCTGCCGTCGACTGTCCTCGGAAGCGGCGGAGTTCTTTACAGTAAGCACCATCGGACCTGATGGCTGACTGAGAAGCCACAGTCAGCCATATCGCGTGTGGCTCAAGCCTTAATCCCGTTTAACCAATCCGGTCAGCACCGGACGTTAATGGAAGGAACGGTCTTAATGTGACC

>SA11 NSP5 high GC

GGCTTTTAAAGCGCTACAGTGATGTCTCTCAGTATTGACGTGACGAGTCTTCCTTCTATTCCTTCAACTATATATAAGAATGAATCGTCTTCAACAACGTCAACGCTGAGCGGCAAAAGCATCGGCCGCAGCGAACAGTATATCAGCCCGGATGCGGAAGCGTTTAACAAATATATGCTGAGCAAAAGCCCGGAAGATATCGGCCCGAGCGATAGCGCGAGCAACGATCCGCTGACGAGCTTTAGCATCCGCAGCAACGCGGTGAAAACGAACGCGGATGCGGGCGTGAGCATGGATAGCAGCGCGCAGAGCCGCCCGAGCAGCAACGTGGGCTGCGATCAGGTGGATTTTAGCCTGAACAAAGGCCTGAAAGTGAAAGCGAACCTGGATAGCAGCATCAGCATCAGCACGGATACGAAAAAAGAAAAAAGCAAACAGAACCATAAAAGCCGCAAACATTATCCGCGCATCGAAGCGGAAAGCGATAGCGATGATTATGTGCTGGATGATAGCGATAGCGATGATGGCAAATGCAAAAACTGCAAATATAAAAAAAAATATTTTGCGTTAAGAATGAGAATGAAACAAGTCGCAATGCAATTGATTGAAGATTTGTAAGTCTGACCTGGGAACACACTAGGGAGCTCCCCACTCCCGTTTTGTGACC
